# Research on Artificial-Intelligence-Assisted Medicine: A Survey on Medical Artificial Intelligence

**DOI:** 10.3390/diagnostics14141472

**Published:** 2024-07-09

**Authors:** Fangfang Gou, Jun Liu, Chunwen Xiao, Jia Wu

**Affiliations:** 1 State Key Laboratory of Public Big Data, College of Computer Science and Technology, Guizhou University, Guiyang 550025, China; gff8221@163.com; 2 The Second People’s Hospital of Huaihua, Huaihua 418000, China; markliu8899@163.com; 3 Research Center for Artificial Intelligence, Monash University, Melbourne, Clayton, VIC 3800, Australia

**Keywords:** artificial intelligence in medicine, assisted diagnosis, genomics, drug development, medical imaging, health care management

## Abstract

With the improvement of economic conditions and the increase in living standards, people’s attention in regard to health is also continuously increasing. They are beginning to place their hopes on machines, expecting artificial intelligence (AI) to provide a more humanized medical environment and personalized services, thus greatly expanding the supply and bridging the gap between resource supply and demand. With the development of IoT technology, the arrival of the 5G and 6G communication era, and the enhancement of computing capabilities in particular, the development and application of AI-assisted healthcare have been further promoted. Currently, research on and the application of artificial intelligence in the field of medical assistance are continuously deepening and expanding. AI holds immense economic value and has many potential applications in regard to medical institutions, patients, and healthcare professionals. It has the ability to enhance medical efficiency, reduce healthcare costs, improve the quality of healthcare services, and provide a more intelligent and humanized service experience for healthcare professionals and patients. This study elaborates on AI development history and development timelines in the medical field, types of AI technologies in healthcare informatics, the application of AI in the medical field, and opportunities and challenges of AI in the field of medicine. The combination of healthcare and artificial intelligence has a profound impact on human life, improving human health levels and quality of life and changing human lifestyles.

## 1. Introduction

In recent years, the application of artificial intelligence in various fields has rapidly spread. People’s demand for health improvements continues to increase, promoting the development of artificial intelligence (AI) in the field of complementary medicine [1]. In regard to the problems of lack of medical resources, shortage of medical technology, and uneven regional distribution of medical resources, people have begun to pin their hopes on machines [2,3,4]. More and more people hope that artificial intelligence can ease the growing pressure on hospitals and improve medical treatment capacities.

Artificial intelligence involves a machine or computer training a model by simulating a large amount of data so that it can learn [5]. The purpose of learning is primarily to achieve machines that can simulate human behavior and demonstrate human intelligence. According to a great number of texts, AI may learn from experience and simulate human thinking to make quick responses and rational decisions with decision-making systems [6]. AI is widely used in various technologies and fields. It involves a series of learning methods, including, but not limited to, machine learning, natural language processing (NLP), deep learning, representation learning and reinforcement learning, heuristic analysis, etc. [7].

With the development needs of China’s medical and health care industries, as well as the rapid development of the Internet, big data, cloud computing, and other related technologies, the scope of research and application of artificial intelligence in the field of assisted medical care has been expanding in terms of depth and breadth [8,9]. AI can not only alleviate the problem of missed diagnosis and misdiagnosis, make up for the gap between supply and demand of resources, and provide health consulting services, but it can also promote the speed of drug research and development and genetic testing, improve the efficiency of pharmaceuticals and the accuracy of surgical operations, improve the cycle management of patient prevention and diagnosis, etc. [10,11]. AI is a key technology for demonstrating precision medicine. It has great economic value and application scope for both medical institutions and both patients and doctors. It runs through every link before, during, and after diagnosis, and achieves penetration in every link [12].

In terms of pre-diagnosis prevention, some large-scale epidemics, such as the Sars virus, Ebola virus, and COVID-19, it is challenging to mobilize a large number of healthcare workers for genetic sequencing in a short timeframe; AI enables more professional and efficient gene sequencing and detection. Traditional genetic testing methods face the problems of huge gene arrays, time-consuming, labor-intensive, and high-cost manual experiments, and low accuracy [13]. AI uses its powerful computing power to complete accurate data analysis and information integration between different databases [14,15,16]. It can mine deep-level association structures and discover potential links between mutation sites and diseases, thereby providing more accurate and faster disease prediction and analysis results and realizing personalized and precise disease intervention programs [17,18,19,20,21].

The application of AI in drug research can achieve the purpose of shortening the time and cost of drug development [22,23,24]. The application of AI in drug mining mainly includes new drug research and development, new use of old drugs, drug screening, drug side effect prediction, drug tracking research, etc. [25,26,27]. The use of big data analysis and other technologies to analyze drug structure and drug efficacy can greatly shorten the time of drug development, reduce costs, improve the accuracy of drug development, and make it possible to develop personalized treatment drugs [28].

When making diagnostic and treatment judgments, AI-assisted medicine enables more accurate and efficient assisted diagnosis. Traditional manual detection of medical images faces a shortage of specialized physicians and a large amount of patient data, in which challenges such as uneven supply and the large number of images viewed or interpreted by physicians become more prominent [29,30,31]. For traditional manual detection of medical images, there are problems such as an unbalanced supply, insufficient number of doctors, and a large number of reading images [29,30,31]. AI uses its computer vision technology to realize lesion identification and labeling, image segmentation, feature extraction, quantitative analysis, comparative analysis, adaptive radiotherapy, 3D reconstruction, etc. [32,33,34,35], to provide assistance and reference for doctors’ clinical examinations [36,37,38]. In addition, due to the inconsistency of data standards, the large amount of data, unstructured data, and other problems, the manual integration and application of electronic medical record data is extremely difficult [39,40,41,42,43]. AI can realize management analysis, data extraction, potential information mining, and prediction of disease risk in a huge patient medical record database [44,45].

In terms of post-diagnosis and treatment, AI promotes health management to become the first choice for a healthy life in the future [46,47]. On the one hand, the traditional medical path of “treatment after illness” is gradually transformed into prevention in advance to cut off the root cause of illness and reduce the probability of illness [48,49]. On the other hand, AI uses data processing to evaluate the overall state of individuals, design personalized health plans, and establish health records to remind users to pay attention to their physical health [50,51,52]. It mainly includes the rationalization of personal dietary structure, physical health management, and attention to psychological state.

Additionally, the application of AI in medical robots is gradually spreading. Robotic technology has been widely used in many scenarios such as surgery, image positioning, rehabilitation training, nursing services, consultation, and hospital logistics [53,54,55]. It unlocks people’s hands while improving healthcare (by using AI technology to free up the hands of healthcare professionals, they can apply their skills and expertise more effectively to improve healthcare delivery). A surgical robot can perform precise resection and undertake the repair of lesions [56]. Rehabilitation robots are an important means to cope with the aging population and the growing demand for medical resources [57]. Non-surgical diagnosis and treatment robots can help read images, evaluate treatment effects after surgery, and improve diagnosis and treatment accuracy [58]. Service robots can provide high-precision, high-intensity, and long-term medical services, freeing doctors from complex, tedious, and repetitive labor [59,60].

The first chapter of this study first elaborates on the development of artificial intelligence and its current status in the field of assistive healthcare. Secondly, Section 2 integrates artificial intelligence technology, frameworks, and AI technologies used in the medical field. Then, Section 3 provides an overview of the research and applications of artificial intelligence in gene detection, drug discovery and development, medical imaging, electronic medical records, health management, and robotics, comparing the advantages of various research methods using different technologies. Section 4 analyzes the challenges and opportunities faced by medical artificial intelligence, and, finally, Section 5 provides a summary, analysis, and outlook on the application and development of artificial-intelligence-assisted healthcare.

### 1.1. The Development History of Artificial Intelligence

In the 60 years or so since John McCarthy coined the term “artificial intelligence” at the Dartmouth Conference in 1956, the development of AI has not been smooth sailing. It has experienced the Pre-AI era, the first trough, the second boom, the second trough, and other periods.

**Figure 1:**
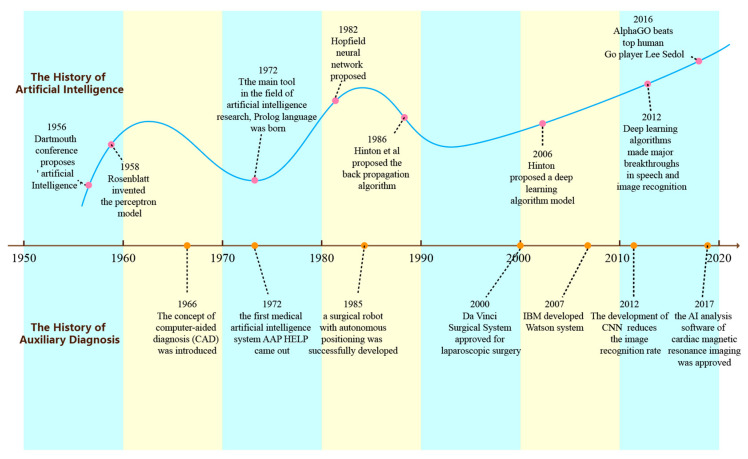
AI development history and development timeline in the medical field.

In the first golden period of development, the first truly excellent artificial neural network, the perceptron, was produced. Minsky and S. Papert published the book “Perceptron”, and, in 1972, the Prolog language, the main tool in the field of early artificial intelligence research, was born [61]. Due to the criticism of AI from all sides and the lack of research funding, AI development stagnated and soon entered a low period. In particular, Minsky’s criticism of the perceptron was almost devastating to the development of neural networks, leading to the disappearance of neural networks for nearly 10 years. It was not until the 1980s that “expert systems” began to be accepted globally, and knowledge processing became the focus of the AI field. In addition, Japan and the United States should began to invest more in AI, and the AI field ushered in another recovery. A new type of neural network proposed by John Hopfield in 1982, now known as the Hopfield network, brought new life to the silent neural network. The most widely used back propagation (BP) network was also born during this period. Because the expert system can only deal with problems in specific fields and the maintenance cost is high, AI soon fell into a low period again. During this period, the market demand for AI dropped significantly, and the financial crisis the industry faced at this stage was more serious. In recent years, with the development of deep learning, AI has risen again and entered a prosperous period. In 2016, Google’s Alpha Go competed with the international Go champion and won by a ratio of 4:1, marking the point where AI technology has matured and entered our lives. The upper part of Figure 1 shows the process of AI development. 

### 1.2. The Development of AI in the Medical Field

In the past few decades, AI has experienced ups and downs and has made tremendous progress and developments. In the 1970s, AI started to explore applications in the medical field, as shown in Figure 1. In 1972, the AAPHelp system, developed by the University of Leeds, which can assist in the diagnosis of severe abdominal pain and predict the need of surgery, was the earliest breakthrough of AI in the medical field. Subsequently, the rule-based AI decision-making system was used for the analysis of electrocardiograms (ECGs) during the clinical diagnosis of patients, and the INTERNISTI system was used for auxiliary diagnosis of complex diseases in internal medicine. However, rule-based methods require medical experts to construct specific rules; the system is expensive to construct, poorly maintained, and its performance is limited by prior medical knowledge.

Despite the shortcomings of rule-based methods, AI systems at this stage have also achieved varying degrees of clinical application. Subsequently, the rise of neural networks further promoted the development of AI in the medical field. Machine learning transforms real-world complex medical problems into mathematical problems by eliminating the need to consider complex input feature interactions. It drives the development and application of medical applications and becomes the framework of choice for building medical AI [62]. With the development of big data and the continuous improvement of computing power, deep learning is being widely used in the medical field. Deep neural networks have achieved breakthroughs in electronic medical records and physiological signals through their superior data fitting capabilities. In particular, the ability of medical image recognition and analysis based on deep learning far exceeds that of humans. The U-Net network does not require a large amount of training data like convolutional neural networks (CNN) and has achieved good application results in many specific scenarios.

At present, with the development of big data, the Internet, and hardware technology, AI has begun to be widely used in the field of smart medical care. Strategies such as “big health”, “precision medicine”, and “medical big data” have been proposed around the world, further promoting the development and application of AI in the field of smart medical care. In 2018, more than 100 AI medical start-up companies have been established around the world, such as IBM’s Watson Health, the Tencent Miying Medical Laboratory, and iFLYTEK’s Smart Medical Division. In recent years, this number has continued to increase.

### 1.3. Status Quo of AI in the Field of Assisted Medicine

The field of AI-assisted medicine is constantly expanding. On the industry side, AI healthcare-related investments are on the rise. Identifying pain points and maximizing the value of AI to human beings are key issues in the sustainable development of the medical industry. At present, more than 90% of medical institutions in the world have begun to plan AI strategies. In 2025, the total value of AI in the global application market is expected to reach USD 127 billion, and the medical industry is expected to account for 20% of the AI market. In China, medical AI is developing rapidly. In 2021 the scale of China’s artificial intelligence healthcare market was close to CNY 6 billion, and it is expected that, by 2025, the overall scale of AI medical care will reach CNY 38.5 billion. Investments in AI medical R&D are also continuing to increase. In 2015, U.S. investment in AI-related R&D reached USD 1.1 billion. The global healthcare AI market is expected to be worth USD 3.6 billion in 2025, indicating an annual growth rate of 50% compared to USD 2 billion in 2018.

At the same time, scientific research achievements in the research and development and application of AI-related technologies have doubled in the past ten years. In the ten years from 2011 to 2022, 46 top conferences in the field of AI would receive 185,241 papers, and 258,268 authors would participate in these conferences. There were only 162,444 AI-related journal papers in 2010, and this amount grew to 334,497 in 2021. Among them, China continues to lead the world in the number of AI journals, conferences, and knowledge base publications. In terms of journal articles, China has the largest number of published papers, accounting for 27.6% of the world’s journal AI publications. In addition, as of 2020, there are 126 research institutions engaged in AI-related clinical trials in the world, of which China accounts for 21.4%, reaching 27. The research institutions with the largest number of clinical trials initiated in the world are also based in China.

The policies promulgated by various countries around the world continue to promote the development of AI. Since 2016, more than 40 countries and regions have placed AI development at a national strategic level. Especially after the new crown epidemic, more and more countries have realized that AI is very important for their international competitiveness. In 2017, China began to emphasize the promotion of AI treatment in the “New Generation AI Development Plan” and establish a fast and accurate AI-assisted medical system. In 2021, in the “14th Five-Year Plan” outline, it is emphasized that the deep integration of AI will be promoted through the Internet, big data, and other industries. Technology development and incentive policies have contributed to the rapid development of AI-assisted healthcare in China, with rising industrial competitiveness and increasing research and development (R&D) capabilities. AI-assisted medical care is constantly changing the development of China’s medical industry.

In general, the rise of the Internet industry, the development and change in hardware technology, the rapid advancement of computing power, and the continuous enrichment of data have promoted the development and application of AI in the medical field.

## 2. AI in Healthcare Informatics

Artificial intelligence has been widely used in research in various fields of medical informatics, such as AI medical imaging, AI medical robots, AI pharmaceuticals, electronic medical records, and so on. However, today’s artificial intelligence is still dominated by weak artificial intelligence. Weak artificial intelligence systems are based on algorithms, usually using machine learning and deep learning theories to analyze complex problems [49]. To better help readers understand the field of global artificial intelligence medical assistance, we introduce the methods and processes of artificial intelligence in detail in this chapter.

### 2.1. Classification of Artificial Intelligence

**Figure 2:**
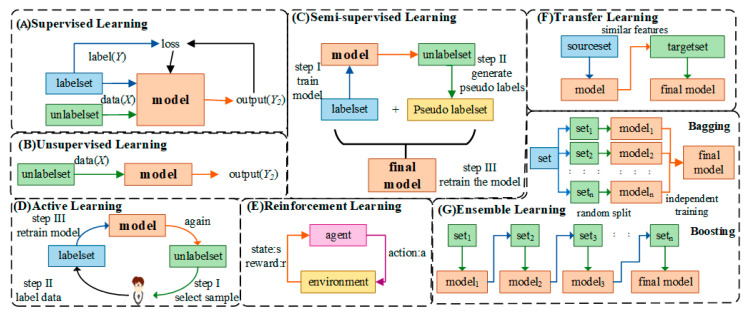
Seven common types of artificial intelligence technologies.

In the application of medical AI, some algorithms are often used to process and analyze data. Table 1 summarizes some commonly used algorithms in the AI field and their advantages and limitations. In addition, these AI algorithms used for medical assistance are generally divided into the following seven categories:

Supervised Learning: This is the most conventional and most used class of AI algorithms. As shown in Figure 2A, it first requires a sufficient amount of input data (usually a vector, and possibly an image matrix; X) and the corresponding ground-truth output (or label; Y), and it then extracts from the data the input-to-output mapping function: $Y=W\left(X\right)$.

Unsupervised learning: Compared with supervised learning, it does not require labeled data, i.e., the input data does not contain labels. Instead of responding to feedback, it addresses various problems in pattern recognition based on commonalities in the data, as shown in Figure 2B. In medical scenarios, a common problem is the high cost of manual labeling, and unsupervised learning can replace manual labeling or classification.

Semi-supervised learning: This is a class of algorithms combining supervised and semi-supervised learning. In its input data, there is a small amount of labeled data and more unlabeled data, as shown in Figure 2C. Semi-supervised learning algorithms can obtain high-accuracy results because they only require a small amount of labeled data.

Active Learning: This is a special semi-supervised learning algorithm, as shown in Figure 2D. The purpose of active learning is to reduce unlabeled data and gradually improve the performance of the algorithm by manually labeling the most informative unlabeled data intelligently selected by it.

Reinforcement learning: As shown in Figure 2E, this is derived from the behaviorism theory in psychology, which emphasizes that the agent will produce habitual behaviors that can obtain the greatest benefits under the stimulation of rewards or punishments given by the environment.

Transfer learning: As shown in Figure 2F, its core idea is to use existing knowledge to learn new knowledge by finding the similarities between existing knowledge and new knowledge.

Ensemble learning: As shown in Figure 2G, this basic process is used to construct multiple sub-optimal learners and then combine them to form an integrated learner with stronger generalization ability. Well-known ensemble algorithms include bagging and boosting.

### 2.2. Artificial Intelligence Frameworks

There are many frameworks based on AI at present, and because each framework focuses on different focuses, it varies according to different needs, such as computer vision, natural language processing, etc. Therefore, the following mainly introduces the current mainstream artificial intelligence framework. Many of these frameworks are currently being maintained by software developers on an ongoing basis, and most new discoveries are quickly incorporated into them. While a proper graphics processing unit (GPU) is required to take full advantage of these modern frameworks, most frameworks also provide Central Processing Unit (CPU) support for training and testing small models. These frameworks allow their users to directly test different network architectures, their hyper parameter settings, etc., without actually having to perform the tasks the layers undertake, as well as the algorithms that train them. These layers and associated algorithms are pre-implemented in the framework library. Below, we list the currently popular deep learning frameworks, as shown in Table 2.

### 2.3. Modeling Methods of Artificial Intelligence

**Figure 3:**
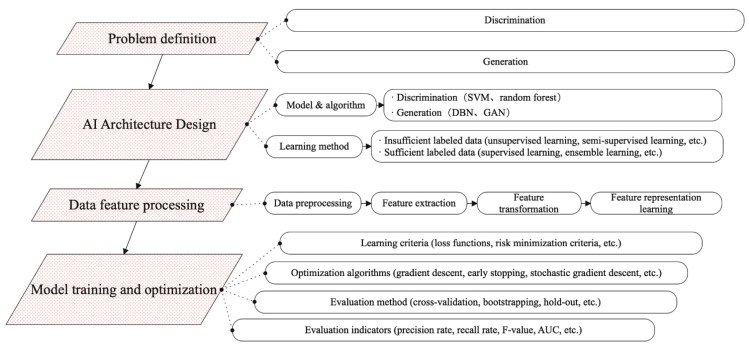
AI modeling process.

When applying an AI model, the first thing to consider is what kind of problem you want to solve and what kind of results you want to achieve. Depending on the problem defined, different models are used to solve it. To apply AI models in the medical field, it is also necessary to select an appropriate learning method according to the input data. For example, the labeling of medical data relies too much on expert knowledge and past experience. The labeling process is time-consuming and labor-intensive, and it does not have scalability and generalization capabilities. Therefore, when building a model, it is often found that there is little or no labeled data. Then, you can consider using unsupervised learning, semi-supervised learning, reinforcement learning, or transfer learning to solve the problem. Artificial intelligence methods have the potential to add soft tissue navigation during surgery to highlight risks and target structures, effectively estimating non-rigid deformations of internal organ structures. Figure 3 shows the process of artificial intelligence modeling:

## 3. The Application of AI in the Medical Field

Artificial intelligence plays an indispensable role in the medical field. So far, AI has been involved in assisted medical care in a variety of ways, realizing the full penetration of a wide range of applications such as disease-assisted screening, clinically assisted decision-making, drug-assisted research and development, and mobile medical health management. It creates a convenient and efficient medical experience for patients and improves the efficiency and economic benefits of medical organizations. This chapter focuses on the six main application directions of artificial intelligence in the medical field, namely genomics, drug research and development, medical imaging, electronic medical records, health management, and AI robots. Examples are given to illustrate the current research status and challenges in these six fields.

### 3.1. The Application of AI in Genomics

**Figure 4:**
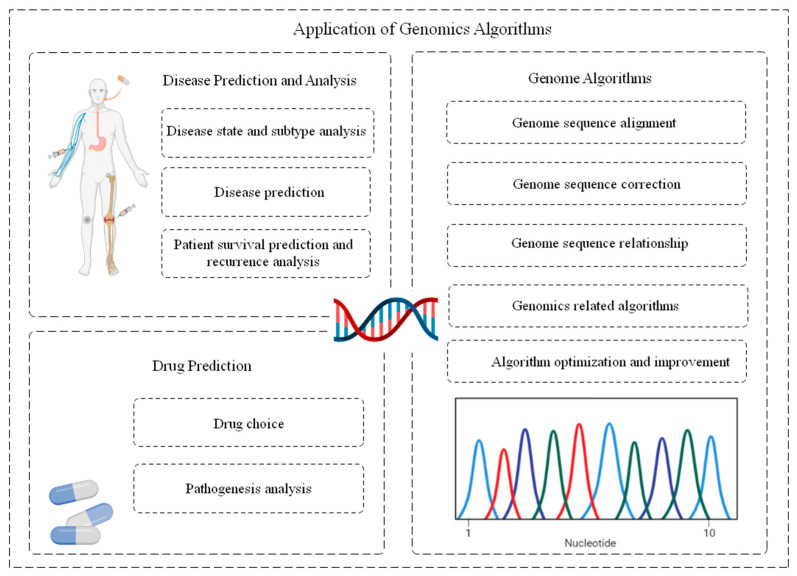
Common Applications of Genomic Algorithms.

With the development and upgrading of modern genome sequencing technology and bioinformatics, various genome applications have been integrated and next-generation sequencing technology (NGS) has been developed, including illumina sequencing, Roche 454 sequencing, and SOLiD sequencing [25]. These technologies bring large-scale genomic data. The amount of common genomic calculation data is about 20–300 GB, and the amount of more in-depth calculation and analysis data will even reach the TB level. Under the demand of efficient computing of large amounts of data, various machine learning and deep learning algorithms applied to genomics have emerged. These algorithms are commonly used for in-depth mining and parsing of various types of information, and they are further used for disease diagnosis, drug selection, genomics algorithms, and many of the following subdivision areas, such as Figure 4. This further shows the broad application prospect of artificial intelligence-related algorithms in medical field.

#### 3.1.1. Disease Prediction and Analysis

In traditional medicine, disease prediction and accurate prediction of patient survival is a very difficult problem. It is necessary to combine the rich experience of doctors and the specific clinical manifestations of patients with high unpredictability and misjudgment rates. The genomics data analysis method can make use of individual customized biological genetic data in regard to patients with diseases to realize the classification of disease subtypes and the survival prediction of patients, which can solve the existing problems to some extent. The analysis results have important guiding significance for the formulation of treatment plans and the selection of medical means for patients.

**Figure 5:**
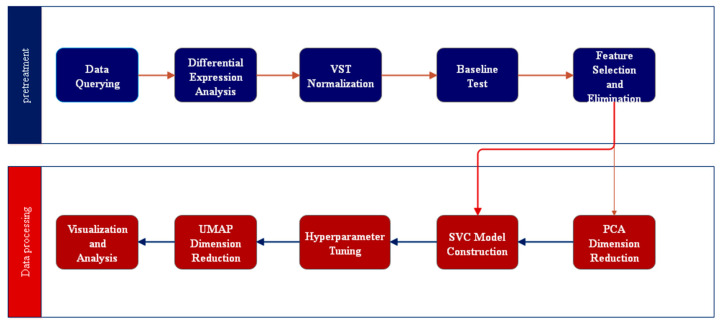
Workflow of Classification Algorithm for Disease Subtypes.

The workflow of the algorithm for disease subtype classification is roughly shown in Figure 5. This kind of algorithm uses the genome data set to analyze the differential expression of genes, extract the classification characteristics, and conduct machine learning to model and train. Finally, the prediction results are obtained and applied to the practical application analysis. At present, the classical classification algorithms include k-nearest neighbor (KNN), support vector machine (SVM), multi-layer perceptron (MLP), and gcForest [13]. Although the classical classification algorithm has stronger robustness, it often has the problems of information loss and relatively low accuracy. To solve these problems, the existing deep learning algorithms mainly improve and improve the disease subtype prediction algorithm in the aspects of data set types and data processing flow. The good results in practical verification also basically meet the requirements of high reliability and high accuracy of medical auxiliary diagnostic methods.

Various deep learning algorithms are widely used in disease prediction. In the use of algorithm data, MicroRNA (miRNA) plays a special role in the pathogenesis of diseases, so it is often used as an important input data in the calculation process. The miRNA-based data algorithm HOPA_MDA [7] extracted miRNA functional similarity and disease semantic similarity from miRNA data and combined them for calculation. The results helped to develop individualized diagnostic schemes, analyze the pathogenesis of diseases, and participate in promoting medical research [63]. But the disadvantage is over-reliance on the initial miRNA data associated with diseases, so there are often insufficient data and lag prediction problems in some new diseases and rare diseases. Based on this problem, RW-RDGN [64], OMMFS [65], and other algorithms turn to use multi-omics data for analysis. Due to its data characteristics, such methods can realize multi-dimensional data analysis and hidden information mining. Multivariate data dilute the dependence on existing information and are more widely used in disease gene prediction. The application of a deep learning algorithm can also be further expanded, such as the use of Magnetic Resonance Imaging (MRI) images and gene data for schizophrenia prediction and immune disease prediction and adjuvant therapy.

In patient survival prediction, the existing computer-aided methods make researchers dispose of most of the tedious tissue structure analysis and physiological analysis. In the method, multi-level information and omics data for patient survival prediction has become the main method of genomic data application. The existing algorithm framework tends to use multi-source data fusion and graph convolution networks [65]. Although these algorithms achieve high accuracy in the prediction results, the repeated calculation caused by the introduction of massive samples in the data makes the algorithm still need some improvement. Aiming at this problem, reference [66] used methods such as the introduction of personalized annotation of gene expression data networks and the simultaneous extraction of various features of multi-layer neural network models to avoid the repeated introduction of samples and solve the problem of low algorithm performance. The application of genomic data reflects, to some extent, the great potential and development direction of artificial intelligence in the field of disease prediction. As shown in Table 3, some algorithms for disease prediction and analysis are listed. It is expected that the future application of genomic data in disease prediction will provide important reference information for medical services.

#### 3.1.2. Analysis of Drugs and Pathogenesis

Under the existing technical conditions, the following can be achieved: using artificial intelligence and its algorithm to build disease similarity network and help drug selection. This is of great significance in clarifying the relationship between the origin of diseases and molecular mechanisms and in studying the function of diseases and medical research. In terms of further subdivisions, genomics analysis algorithms can be roughly classified into three categories [68]:

(1) Reliability interpretability algorithm: including multi-gene property compatible multivariate method, PRS, data-driven multivariate multimode method.
(2) Improve diagnosis algorithm: multi-level and multi-dimensional framework, etc.
(3) Improve treatment algorithms: biomarker prediction algorithms, treatment response prediction, etc.

In terms of function, deep learning algorithms can be used for drug prediction. In the application process, the existing algorithms mainly use indirect methods for drug selection, as follows: The relationship between genes and diseases is first calculated; then the alternative drugs are further calculated. References use a protein–protein interaction network (PPI) and a series of pathway databases to construct disease pathway networks or pathology to identify relevant genes, thus exploring diseases and their internal interactions. Reference for breast cancer disease networks prediction, new disease correlation research and drug selection. The prediction results can also be extended to the establishment of new biological mechanisms related to drug responses to other diseases.

In the process of drug selection and screening by artificial intelligence, the pathogenesis of specific diseases can be further explained by gene regulation mechanisms in the process of drug screening, and the origin of diseases can be revealed. The analysis algorithm of screening drugs using genomic data has been widely used in the exploration and analysis of the pathogenesis of some diseases, including the diagnosis of genetic diseases through integration method and case–control, the interpretation of cancer genome data and the discovery of mutation-driven pathways, and the analysis of the complex pathogenesis of malignant tumors. Artificial intelligence analysis has greatly reduced the research cycle of cancer pathways and pathogenesis. With the effective intervention of technology, the research cycle in the unit of year can even be shortened to a month. It is a fast method for discovering disease-related genes and pathways, which is helpful for the comprehensive analysis of the pathogenesis of diseases. To a certain extent, the genomics pathogenesis analysis algorithm solves the problem of the difficulty in comprehensively analyzing the pathogenesis predicted by genomics data. Because of its easy generalization and diversity in application, it can also be used in diagnostic systems and precision medicine for mental diseases.

#### 3.1.3. Genome Algorithm

**Figure 6:**
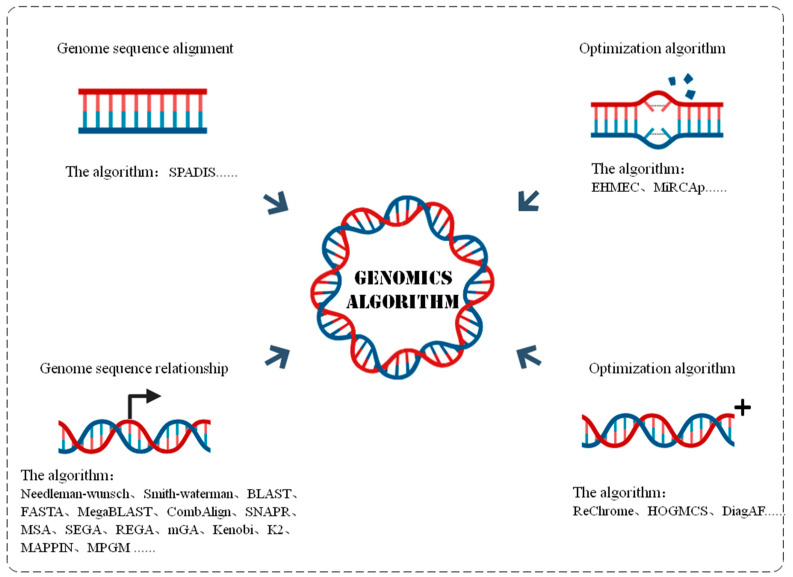
Summary of genomic algorithm.

In the field of bioinformatics, DeoxyriboNucleic Acid (DNA) sequencing data have a wide and important role in seeking to solve the problems in the research, identification, and biochemical fields of genetic diseases. This involves basic steps such as genome sequence alignment, correction, and even alignment. The overview of genomics algorithms is shown in Figure 6.

Genome sequence comparison is a fundamental research issue in genomics. In the sequence alignment process, the genome rearrangement effect will greatly affect the genome alignment efficiency. The existing alignment algorithms are difficult to compare to complex gene sequences, and the computational efficiency and accuracy are difficult to improve. In reference [68], a heuristic algorithm based on local search, genetic algorithms, and greedy random adaptive search process technology was used to study the rearrangement distance between genomes of replication genes, and good sequence alignment results were obtained. In reference [69], the complementary effect means of single nucleotide polymorphism phenotypic interpretation brought into the genome showed a stronger recognition ability and running speed than the existing commonly used algorithms. These methods can further strengthen the algorithmic sequence comparison ability and recognition ability, and they have achieved better results in empirical studies, which are widely used in the fields of disease prediction and patient survival prediction.

In genome sequence modification. The utilization of genomic data will decrease due to the error of genomic data. Reference [70] used the extended heuristic algorithm to correct DNA sequences for the mismatch problem in genome sequence comparison so that the number of errors between DNA reading arrays was greatly reduced. In general, the existing genome sequence correction studies mainly achieve genome sequence correction through the optimization of algorithms and data. However, the optimization of hardware, such as instrument processing for genome sequencing, is relatively small but also effective. In Reference [71], a Minolon Read Correction Algorithm (MiRCA) was used to correct deletion, insertion and substitution errors by inserting multiple sequence alignments into the data reading program of the gene sequencing instrument. This method modifies the sequence from the upstream data acquisition link and then obtains the available data with high quality and high accuracy so that the algorithm can calculate the relevant prediction results and analysis results with high accuracy.

A comparison of the correct genome sequences can be given for the next sequence alignment and genome relationship analysis. An exploration of genome relationships has been applied in the construction of a multi-scale biomolecular system model and the identification and exploration of protein binding regions. The existing sequence alignment techniques can be divided into the following three categories [65,72]: pairwise local and global and local sequence alignment, multiple sequence alignment, and word and structure-based sequence alignment. The first type of global and local sequence alignment, global alignment, is often used to determine the homology analysis between proteins, and local alignment focuses on finding optimization and alignment or closely related fragments between the two sequences. The second type of multiple sequence alignment (MSA) plays an important role in exploring the similarity and relationship between sequences and finding the special motif of sequences. The third type of sequence alignment is based on words and structures: FASTA and the Basic Local Alignment Search Tool (BLAST) are more typical algorithms which can directly approximate alignment and optimize the measurement of local similarities; they are also simple, robust, flexible and easy to handle.

The accumulation and analysis of genomic data makes the screening of disease biomarkers possible. There are more popular supervised learning methods, unsupervised learning methods, and molecular networks for disease biomarker prediction. In reference [73], the definition and comparison framework of gene clustering (COMBING) that was proposed, using a clustering algorithm to generate gene clusters with similar expression profiles and feature selections, has achieved good results in the judgment of tumor types and subtypes. An alignment-based networks construction algorithm (ANCA) is proposed in [74], which not only solves the problem of dynamic network topology changing with time but also accurately predicts the missing target network and expands it in real time. It provides an optimization and supplement strategy for the marker prediction algorithm using molecular networks in unsupervised algorithms. The existing algorithms have been preliminarily applied to the judgment of tumor type and subtype characteristics, and they have made some achievements. An artificial intelligence algorithm using genomics data has broad optimization space and improvement directions.

Algorithm optimization methods can be roughly divided into data processing optimization and algorithm process optimization. In terms of the optimization of the data itself, reference [75] used the recursive convolution neural network to analyze the apparent genome data (ReChrome) to reduce the calculation parameters. Reference [76] achieved low-cost calculation for large-scale analysis of frequent genomes by reducing and optimizing the amount of calculation. In the algorithm flow, for the data processing process, extraction, storage, retrieval, genome data compression, data fast retrieval, and data matching [76] can effectively reduce data search time and improve the efficiency of data collection and processing, simplify the pre-processing process time consumption, and improve computational efficiency.

The accuracy and reliability of the algorithm recognition process is gradually improved and the computational difficulty is reduced. The genomics algorithm shows an increasing generalization ability in different data sets and different practical applications. It has been widely used in disease prediction and personalized medical plans formulation. The application proportion in the field of medical biology research, such as disease pathway analysis and biological marker molecular selection, is also increasing day by day. Related application algorithms are shown in Table 4.

### 3.2. The Application of AI in Drug Discovery

**Figure 7:**
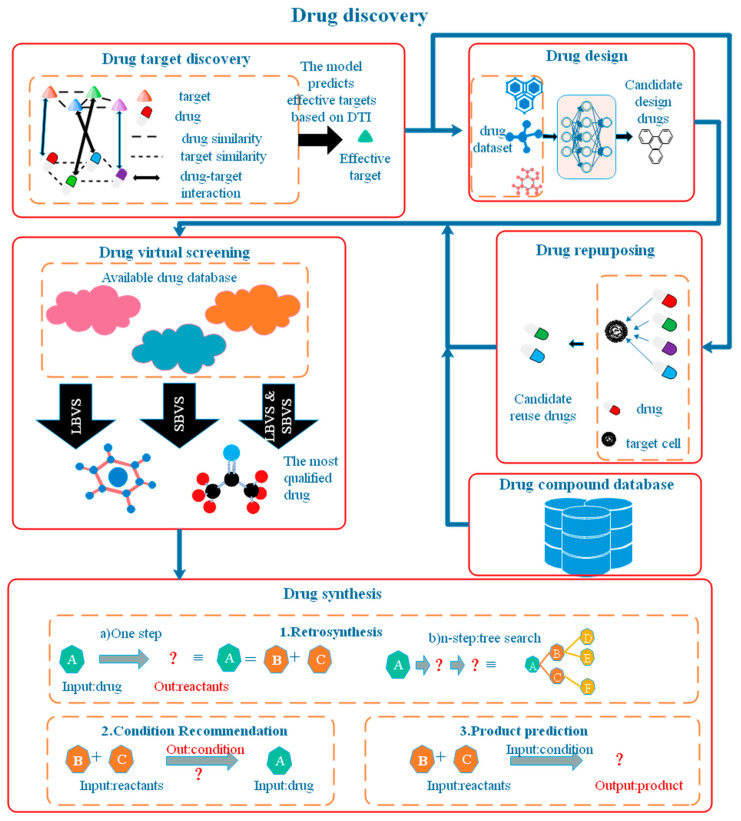
Main components of drug discovery or Drug Discovery.

Another opportunity for the application of artificial intelligence is in the pharmaceutical field. Everyone suffered during the COVID-19 coronavirus pandemic that swept the world in recent years. So far, there are numerous variants of the virus everywhere, and there is still no drug or vaccine that has emerged that can completely cure COVID-19. Everyone is hoping for the emergence of drugs to end the farce caused by COVID-19. Therefore, artificial intelligence is also a hot topic in the field of drug development. One of the most relevant areas involving COVID-19 is drug repurposing. Traditional drug development takes 10–15 years [76], which is too late for the COVID-19 pandemic situation. Drug repurposing, on the other hand, greatly accelerates this process by using existing drugs to treat new diseases. Artificial intelligence can assist humans in drug repurposing. In addition to drug repurposing, artificial intelligence has a wide range of applications in four areas: drug target discovery, drug screening, drug design, and drug synthesis. The various components of drug discovery are shown in Figure 7.

Firstly, in drug target screenings, AI predicts and screens suitable targets through drug–target interactions. Secondly, in drug design, AI can learn previous drug molecule structures and characteristics, and then automatically generate drug molecule structures that meet the requirements. Drug repurposing means looking for the existence of a certain drug among the developed drugs based on the target characteristics of existing diseases and the existing database of alternative drug molecules; the three are combined to establish a database that can be finally used for screening. Third, through drug virtual screening, a large number of unreliable or ineffective drugs are screened out based on molecular characteristics, drug properties, etc. Finally, through artificial intelligence drug synthesis, the three steps of inverse synthesis route prediction, reaction condition prediction, and product prediction are used to determine those compounds that can be synthesized and established with a feasible synthesis route.

#### 3.2.1. Drug Target Discovery

Drug discovery involves many components, and drug targeting, as the first step in drug discovery, is a primary task and daunting challenge for the pharmaceutical and biotechnology industries. The focus of drug target discovery is mainly on the discovery of drug–target interactions (DTIs). The rarity of DTIs is a difficult aspect of drug target discovery. In addition, drug targets may have side effects, such as the unexpected reactions of drugs with non-targeted proteins.

Drug targets can be confirmed by in vitro testing, but this approach is time-consuming and costly. To reduce time and money costs, computer-assisted artificial intelligence methods are gaining attention. Current AI methods for DTI identification are broadly classified into four types: molecular-docking-based, text-mining-based, machine-learning-based, and network-based.

Molecular docking is a simulation-based approach that matches drug molecules based on the 3D structure of proteins. Thus, the method provides an intuitive visual interpretation, but molecular docking simulations are very time-consuming. In addition, it is difficult to obtain the 3D structure of a molecule or protein to scale up to large datasets due to the extreme complexity of the 3D structure. If a protein is unknown, it will not be possible to apply the process. Text mining is a method for processing textual descriptions. It predicts the presence of DTIs by extracting the textual features of the description of a drug-protein. However, due to the diversity of linguistic expressions and the multiplicity of information, the performance of text-mining-based methods is limited as a result. The machine-learning-based approach is to identify DTIs by extracting the biological features of drugs and targets. He et al. [26] proposed the SimBoost method, which is more efficient compared to molecular docking and is text-mining-based. However, the method has two drawbacks: first, the feature representation is restricted to the similarity space, ignoring the rich information in molecular sequences; second, if a completely new molecule is measured, the model will use a relatively unrelated molecule to represent it, which will make the prediction inaccurate. Therefore, the generalization ability of machine-learning-based methods is insufficient in coping with the increasingly large molecular libraries. Network-based methods have become the most popular methods in recent years, of course, due comes from its advantages of high accuracy and efficiency. The deep learning approach is an end-to-end method that eliminates the need for feature engineering, and it automatically extracts useful features from the original sequences of molecules and proteins. Compared to machine learning, deep learning reduces the loss of feature information in prediction DTIs to overcome certain limitations. Öztürk et al. proposed a CNN model that was demonstrated on two publicly available DTI benchmarks [27].

With the advances in hardware and the availability of more and more publicly available biological data in recent years, there are more opportunities for deep learning and drug target discovery. Moreover, in addition to the discovery of existing targets, the latest research directions point to potential new targets for drugs, with the goal of predicting unknown targets and providing new treatments for diseases that can be treated, as well as finding therapeutic options for diseases that are currently difficult to treat, such as cancer and AIDS.

#### 3.2.2. Drug Screening

The traditional screening method involves high-throughput screening, in which a large number of drugs are screened in a single experiment to find useful drugs. As a result, the experimental cost is high. To reduce the cost of drug development, researchers have proposed AI-based virtual screening technology. Virtual screening technology means that, by analyzing the chemical and physical properties of drugs, a large number of unsuitable compounds are screened out from a large number of compounds, and then the few remaining compounds that may meet the requirements are experimentally tested for the activity to screen out the lead compounds. As the number of compounds actually tested experimentally is reduced, the cost is thereby reduced. In addition, the virtual screening takes into account molecular toxicity, etc., which increases the content of the screening.

The existing artificial-intelligence-based virtual screening techniques are divided into two main categories: ligand-based virtual screening (LBVS) and structure-based virtual screening (SBVS). LBVS constructs Quantitative Structure–Activity Relationship (QSAR) models by combining the molecular information of compounds, molecular characterization, and compound types to analyze the relationship between structure and molecular activity, and then screens suitable compounds by the principle of molecular similarity. SBVS is realized by molecular docking technology, which is based on the three-dimensional structure of the receptor. It automatically matches small molecules in the compound database at the binding site and obtains the ranking of compounds through a scoring function.

SBVS has some limitations, such as the difficulty of obtaining the 3D structure of molecules and the difficulty of including protein flexibility in the model. LBVS is highly dependent on the type of molecular characterization and compound class. To further improve the technology of virtual screening, the integration of SBVS and LBVS has become an option in recent years, and the integration is mainly divided into sequential, parallel, and hybrid approaches. Sequential approach integration follows multiple steps, executing the LBVS and SBVS methods sequentially. Parallel execution is filtered by LBVS and SBVS methods simultaneously, taking their common or optimal results. The hybrid approach, on the other hand, transforms LBVS (or SBVS) into the information needed by SBVS (or LBVS) through interaction or similarity docking, truly combining LBVS and SBVS into one independent method. Debnath et al. [28] first explored a pharmacophore model by combining LBVS and SBVS, using a 4.3 × 106 molecule database combined with ADMET criteria to screen the first 500 seedling compounds, which were later evaluated by molecular docking, with the final compounds selected, SD-01 and SD-02, inhibiting the HDAC8 enzyme at IC50 (i.e., inhibitor concentrations yielding half the maximum response) values of 9.0 nM and 2.7 nM, respectively. Wang et al. [77] introduced a combined structure-based virtual screening strategy to address the problem of SBVS neglecting protein flexibility and inaccurate binding affinity prediction, demonstrating the effectiveness of the SBVS combinatorial strategy.

Currently, the ability of deep learning methods to automatically learn complex concepts and high-level abstractions can overcome the limitations of machine learning algorithms and is more promising than machine learning (ML) methods. Therefore, deep learning algorithms are the mainstream of virtual screening nowadays. In addition to the need to expand the application of deep learning algorithms and datasets, sufficiently large databases of drug compounds are inevitable challenges in regard to the development of drug screening. Gentile et al. [78] established a deep docking platform (DD) for virtual screening libraries and achieved a reduction in the range of hundreds to thousands in the number of virtual screening matches by AI methods without significant loss of potential drug candidates. Gonczarek et al. [79] established a new benchmark dataset for SBVS based on three existing databases, DUD-E, MUV, and PDBBind, which are more suitable for training ligands that identify target proteins.

#### 3.2.3. Drug Design

Drug design is the real beginning of drug development, the process of generating new lead compounds with desirable pharmacological and physicochemical properties. The traditional de novo drug design approach is to connect small fragments one by one through growth or genetic algorithms to eventually form the target compound. However, this approach is not well thought out and often requires trade-offs in terms of toxicity, drug properties, absorption, and ease of synthesis, discarding some important properties and thus making the drug somewhat defective. The rise of deep learning offers the possibility to design drugs while considering the drug properties in a comprehensive manner. Algorithms for deep learning include recurrent neural networks, generative adversarial networks, encoders, reinforcement learning, and graph neural networks. However, regardless of the type of model, since most of the evaluation metrics today have their own shortcomings, the models inevitably produce overfitting situations. In addition, existing researchers focus mainly on molecular generation algorithms, with less focus on practical integration.

To make molecular design research more goal-oriented, researchers should collaborate more with laboratories. One, it can design targeted research objectives; two, it can test the goodness of research results through experiments; and, three, it can promote the full automation of drug design from design to fabrication and other processes. Drug design is usually divided into the four steps of design–make–test–analysis (DMTA), and shortening the drug cycle is a major challenge in drug design. The four processes of DMTA typically last 4–8 weeks. Among them, the design and analysis processes are faster, but making is more time-consuming. If the making time can be reduced, then the DMTA cycle time can be significantly shortened. Moreover, 61% of the generated molecules were successfully synthesized in real tests, where 12 novel LXR agonists with low micromolar to submicromolar activity were firmly established. Thus, the automation of the DMTA cycle in drug discovery is expected to be achieved by integrating multiple platforms for each step of drug design.

Moreover, polypharmacology is an innovative idea in drug design. There are multiple factors related to its emergence. One is to investigate drugs that can treat multiple diseases at the same time, thus reducing the cost of drug development. Sorafenib is a small molecule multi-targeted anti-cancer drug. Due to the diverse causes of mutations in cancer, different cancer patients have different causative factors and require different drugs. Sorafenib can be used for the treatment of various diseases such as kidney and liver cancer due to its multi-target design [77]. The second reason is that the disease pathogenesis features are complex and difficult to address through single-target drugs. Multi-target drugs can be effective against multiple targets at the same time, which can largely improve drug treatment effects. Traditional single-target drug studies have repeatedly failed in dealing with Alzheimer’s disease, while there have been several successful cases regarding combination drug therapy and multi-target drug studies against Alzheimer’s disease (AD). Therefore, the study of polypharmacology will likely open a golden era of AD drug research.

The use of artificial intelligence in drug design is a welcome development. In the past, drugs were designed manually through medicinal chemists, but the complexity of drugs that can be designed by human beings has reached its limit, and the fact that diseases such as cancer have not been overcome so far is the best harbinger of this. The emergence of artificial intelligence can shorten the time of drug design and break through human limitations, which is expected to solve the dilemma of drug design.

#### 3.2.4. Drug Synthesis

Artificial intelligence in drug design is not only faster than manual design but also extremely accurate in multiple methods. In the DMTA process, drug design is only the first step, and the second step is drug synthesis. However, deep learning-based algorithms usually do not guarantee the validity of the generated molecules and may present many unrealistic results.

To facilitate the development of deep learning molecular design, the problem of synthesis ability must be addressed. There are currently three main functions of artificial intelligence in drug synthesis: inverse synthetic design, condition recommendation, and product prediction.

Inverse synthesis design involves starting from a target molecule, working backward to deduce which submolecules (precursors) are needed to synthesize that molecule, and then iterating until all the synthesized molecules are marketable chemical materials [23]. This iterative process is difficult for the human brain to cope with. However, computers are very good at iterative problems. With a suitable molecular synthesis capability evaluator, the computer can quickly filter the suitable targets among the thousands of precursors that can be generated at each step and finally design a suitable synthetic route. With a synthetic route, one has to consider whether the designed route is feasible, which requires the determination of the reaction conditions. Unsuitable temperatures, catalysts, etc. can produce a large number of by-products and even prevent the synthesis of the target molecule. Collection of previous experimental data [23]. The construction of an automatic recommendation system for the reaction conditions will greatly facilitate drug synthesis. Finally, there is product prediction, which predicts the products and virtually simulates the drug synthesis process based on the synthesis route and reaction conditions to ensure the operability of the synthesis route. The three steps are intertwined to finally ensure that the synthetic routes designed by artificial intelligence are effective.

The goal of artificial intelligence applied to drug synthesis is not to replace chemists but to assist them. Artificial intelligence can perform tasks that do not require much intellectual and repetitive labor, thus freeing up chemists and giving them more time and energy to work on rare compounds and complex reactions.

#### 3.2.5. Drug Repurposing

The development cycle of new drugs is extremely time-consuming and expensive, typically taking 10–15 years and USD 800 million to USD 1.5 billion. Yet, even so, the success rate for developing new molecular entities is only 2.01%. Drug repurposing can greatly accelerate the drug development process and reduce costs by identifying existing drugs that play a role in various other diseases and using existing drugs to treat new diseases.

Accelerating the process of drug development through drug repurposing can, on the one hand, solve the problem of unmet needs for the treatment of various difficult diseases. On the other hand, it can open up markets, extend the patent term of drugs, and increase revenue sources. In general, drug repurposing has a shorter development cycle and involves less investment and lower risks in the drug discovery process [80].

In fact, because of the diversity and complexity of drugs, it is difficult for researchers to grasp the properties and clinical effects of the various drugs in the drug library, and drug repurposing is therefore not as easy to apply in practice. For example, successful drug repurposing is not common in the precision treatment of oncology. However, the advent of artificial intelligence techniques has greatly improved this situation.

The rise of artificial intelligence technologies is not accidental. In the past, drug repurposing tasks mainly used traditional machine learning algorithms to predict molecular properties, etc., to screen candidates. However, machine learning algorithms require the manual construction of features, descriptors, etc., and can only handle fixed-size inputs, which is highly unscalable. As the demand for drug development becomes more and more voluminous, machine learning algorithms are somewhat inadequate in regard to solving the problem. Meanwhile, artificial intelligence has achieved remarkable success in areas such as natural language processing and computer vision.

There are many precedents of deep learning or machine learning methods being used to facilitate drug repurposing, with the COVID-19 coronavirus sweeping the world being the most widely known. To rapidly develop antiviral drugs and vaccines against COVID-19, artificial intelligence has been used to train, based on existing databases, to predict the clinical properties of drugs and thus enable drug repurposing [80,81]. There are also many other cases. Gysi et al. [80] used artificial intelligence techniques, combining network diffusion and the nearest neighbor algorithms, to rank drugs and predict the top-ranking algorithms, obtaining a 62% success rate. Rajput et al. [81] used machine learning algorithms such as k- tight neighbors, random forests, and deep learning, using “DrugRepV “ repository data for training and validation, and they were able to predict drug repurposing more robustly. The algorithms for drug reuse can be broadly classified into three categories: supervised learning, unsupervised learning, and semi-supervised learning, as shown in Table 5.

Deep learning in drug repurposing, although a small success, currently has many challenges. Similar to drug design, for example, insufficient data and poor data quality are the most important elements that hinder the development of deep learning for drug repurposing. In addition, there are also problems, such as insufficient validation experiments for drug repurposing prediction results. In the future, the establishment of structured and integrated large drug development databases is important in regard to facilitating the development of drug repurposing.

Although AI approaches have shown good performance and promise in the field of drug development, they are still in their initial stages, and the biggest obstacle to development is data. Algorithms are data-centric techniques; therefore, whether it is an artificial intelligence approach in drug screening, drug design, or drug synthesis, adequate training data learning is required for excellent performance and even the ability to push the limits [81]. However, deep learning data for drug development has problems such as multiple sources and heterogeneity, and data is not shared across companies. All these reasons together lead to insufficient data. It is good to note, however, that there are many interested parties who have started to build open datasets for drug development for research purposes.

### 3.3. The Application of AI in Medical Imaging

**Figure 8:**
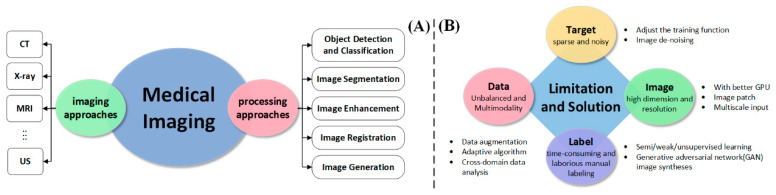
(**A**) Two independent areas of medical imaging. (**B**) Difficulties and solutions encountered in AI-based medical image processing technology.

For medical research, medical imaging is a visual technology and process for generating images of tissues inside the human body in a non-invasive manner. It has two independent domains: medical imaging technology (MIT) and medical image processing technology (MPT). Among them, MIT applies natural physical phenomena (see Figure 8A), such as using light (optical correlation tomography (OCT)), sound (ultrasound (US)), magnets (Magnetic Resonance Imaging (MRI)), rays (X-ray, computed tomography (CT)), and so on [82,83,84,85,86]. They are an essential basis for clinical analysis and judgment of treatment effect.

In MPT, AI uses computer vision technology to optimize and assist in analyzing medical images, which provides more of a basis and quantitative analysis for doctors to assess patient status. Image enhancement, object detection and classification, image segmentation, image registration, image generation, and feature extraction are general MPT methods [87]. AI can improve the quality of images to help radiologists and doctors have a better reading experience. Furthermore, depending on image processing or deep learning algorithms, AI can also automatically or semi-automatically identify lesion areas in medical images, which assists doctors in detecting small lesions (such as lung cancer with high mortality) in their early stages and improves the five-year survival rate of patients [88,89,90]. At the same time, accurate AI-assisted annotation also reduces the heavy workload of doctors.

Given that AI has been widely used in many medical imaging domains, this section cannot cover all domains involving medical imaging in depth. This section focuses on the challenges and the corresponding solutions in the development of AI-assisted MPT in recent years (see Figure 8B). Moreover, we will focus on describing three AI-medical imaging domains (object detection and classification, image segmentation, and image registration) and briefly describe developments in other domains as well. This section is intended to be a clear demonstration of the current research status and highlights of artificial-intelligence-assisted MPT (Modeling and Predictive Techniques) for the readers.

#### 3.3.1. Detection and Classification

Object detection and classification is an important research field in MPT. It can provide specific location and disease details for doctors. Its accurate diagnosis reduces the workload of doctors and has a substantial auxiliary role for doctors in regard to analyzing images.

Unlike natural images, medical images (e.g., MRI, CT, X-ray, etc.) are usually grayscale images, and there are two-dimensional slices and three-dimensional voxels. In addition, many lesions are only small areas and resemble surrounding tissue on images. Traditional medical detection and classification usually use traditional machine learning algorithms and hand-designed extracted features, but they are limited by prior knowledge. With the further development of AI theory and hardware, CNN-based deep learning approaches have become mainstream. In the following, we introduce a combination of medical backgrounds and recent advances in vision algorithms.

**Figure 9:**
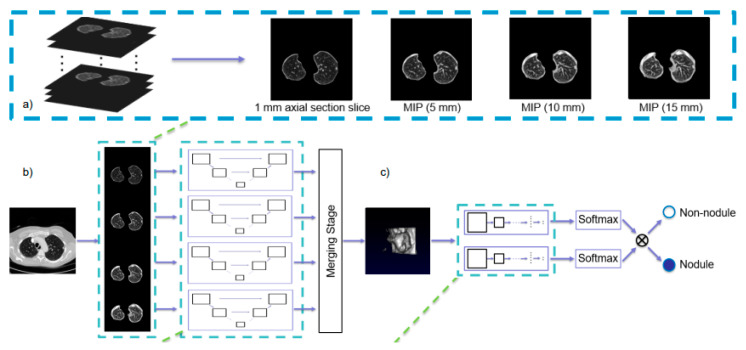
Architecture of a multi-stage 2.5D network for detecting lung nodules [92]. (**a**) is the maximum intensity projection image of axial slices of different thicknesses; (**b**) is the image merging process; (**c**) is the classification process.

In medical imaging, the detection and classification (recognition) of objects are usually staged due to the large image resolution. First, the region of interest (ROI, 2D) or volume of interest (VOI, 3D) is extracted through the detection algorithms and then input into the 2D/3D-based algorithms to output the classification result. Reference [91] proposed two-stage deep learning models for detecting and classifying breast masses in X-ray images. They both use the Yolo detector with the CNN-based classifier and have good performance on the DDSM dataset. Zheng et al. [92] proposed a CNN-based two-stage lung nodule detection algorithm for CT images, as shown in Figure 9. The algorithm first inputs the maximum intensity projection images of axial section slices in different thicknesses (1 mm, 5 mm, 10 mm, 15 mm). This data processing method has better performance in distinguishing nodules and vessels. The detection results are then input into a 3D CNN for false-positive reduction.

Furthermore, some algorithms abandon the multi-stage framework with error propagation and use end-to-end networks that are easy to train. Liu et al. [31] proposed a deep multi-task learning approach to detect pulmonary nodules in CT images. It has lung parenchyma segmentation and nodule detection, where segmentation is used as an attention module to better detect nodules. Reference [93] constructed a densely connected 3D CNN to train the network in an end-to-end multi-task joint method. This approach had high detection accuracy on large-scale CT image lung nodules datasets, such as LUNA and LIDC. Moreover, to elucidate the ambiguous paradigm of tiny object detection, it conducts a further analysis of these networks.

Due to the lack of sufficient annotations, appropriate pre-trained models are essential to classification performance because they have similar data distributions. Therefore, transfer learning (TL) has made outstanding contributions to improving medical image detection performance. Sekhar et al. [36] used a TL model to classify brain tumors in MRI images into three categories (glioma, meningioma, and pituitary). The experiments showed that the pre-trained GooleNet outperformed existing models in the brain tumor classification. Ayana et al. [94] proposed an approach based on a pre-trained model (ImageNet dataset) and a Patch classifier, which can identify breast masses (benign or malignant) in X-ray images. Compared with other Patch-based approaches in the INbreast dataset, its test accuracy is improved by 8% (91.41%/99.34%), which is statistically significant, as shown in Table 6.

#### 3.3.2. Medical Image Segmentation

Image segmentation is a hot topic in terms of image understanding and important medical image processing technology. Image segmentation divides the whole image into several regions, and the elements in a region have similar properties. In medical imaging, image segmentation can extract regions of interest (ROIs) (possibly lesions) to separate background and tumors. It can visualize and quantitatively analyze patient tumors, which is of substantial help to physicians in assessing patients’ status and tumor staging.

With the development of machine learning and deep learning, the performance of AI for medical image segmentation has been continuously improved. However, image segmentation for medical imaging still faces two problems:

(1) Unlike natural images, medical images have blurred discontinuous boundaries, which can lead to misidentification of adjacent regions.
(2) Medical images often have few annotations, and how to use a large number of unlabeled data is the direction of image segmentation development.

As for blurred discontinuous boundaries, in recent studies, some algorithms focus on optimizing segmentation results according to the image grayscale. Wu et al. [37] presented a novel algorithm for segmenting nodules in chest CT images. The algorithm uses a 3D Conditional Random Field (CRF) to continuously optimize the training set during the training phase, and the optimized data accelerate the convergence of the 3D-UNet model and reduce the training time. In addition, it is also more meaningful for optimal segmentation of adhesive pulmonary nodules (parasternal and paravascular nodules) and ground glass pulmonary nodules (GGNs) on CT images. Since there is a strict mapping between signed distance maps (SDMs) computed from object boundary contours and binary segmentation maps, the segmentation task is transformed to predict SDMs with better smoothness and continuity in shape. Such algorithms perform as well as current 3D CNNs on the segmentation datasets of multiple organs.

Additionally, there are other studies that focus on adding prior knowledge. Yu et al. [95] added an adaptive intensity look-up table to the segmentation framework which can adapt to newly added MRI images and dynamically transform the intensity contrast of input brain tumor images. Manuel et al. [96] proposed a loss function for the fundus vessel segmentation, whose function considers the distance of each pixel to the vessel tree and finally obtains a probability map of the vessel structure. Usman et al. [97] proposed a semi-automatic fully supervised learning algorithm to accurately extract lung nodules from 3D chest CT images. This algorithm achieves good segmentation performance, but it requires the ROI of a slice, and the results are limited by the ROI labeling.

In regard to a few annotations, semi/weak supervised learning are usually good solutions. Study [98] constructed generative adversarial networks for segmenting breast cancer in X-rays. It effectively uses unannotated images, and with the quality of the labels generated by the generative network being improved, the model could generate different tumors and improve robustness and accuracy. Feng et al. [99] proposed an edge-competitive vessel segmentation network which consists of a segmentation network and two edge-segmentation discriminators. The network uses the two discriminators to distinguish vessels and backgrounds during the adversarial process. The algorithm combines the segmentation task with an edge-aware self-supervision, which is comparable to fully supervised approaches in performance. Furthermore, unsupervised learning is also a feasible research domain. Elham et al. [100] proposed a clustering method for segmenting lung nodules on CT images, which extracted the feature data space from pre-detected nodules and clustered the feature vectors. Gur et al. [101] proposed a novel unsupervised deep learning approach for vessel segmentation in pathological images; it was based on edgeless morphological active contours and could be used with similar datasets. Essam et al. [102] introduced an improved Chimpanzee Optimization Algorithm (ChOA) for extracting and classifying breast cancer regions in infrared images. This algorithm outperformed seven other meta-heuristics (MHs), including the Grey Wolf optimization algorithm, as shown in Table 7.

#### 3.3.3. Medical Image Registration

Image registration is based on the similarity between images as a measure. By looking for a spatial transformation, one image can be mapped to another image, so as to achieve the purpose of information fusion. Among them, we call the image that remains unmoving a fixed image, and the transformed image is called the moving image. In clinical diagnosis, a single modality image does not provide the physician with sufficient information, and it is often necessary to confirm the diagnosis by looking at multiple modalities or imaging multiple times in the same modality. Thus, for the same patient, there may usually be multiple acquisitions of the same image or different images, such as MRIs, CTs, etc. However, this process is entirely based on the spatial imagination and subjective experience of the physician. Therefore, by using image alignment methods, the information from different imaging sources can be accurately fused into the same image to achieve complementary information, making it easier and more accurate for the physician to view the lesion from all angles. At the same time, the alignment of dynamic images acquired at different moments can quantitatively analyze the changes in lesions and organs, making medical diagnosis, surgical planning, and radiotherapy more accurate and reliable.

**Figure 10:**
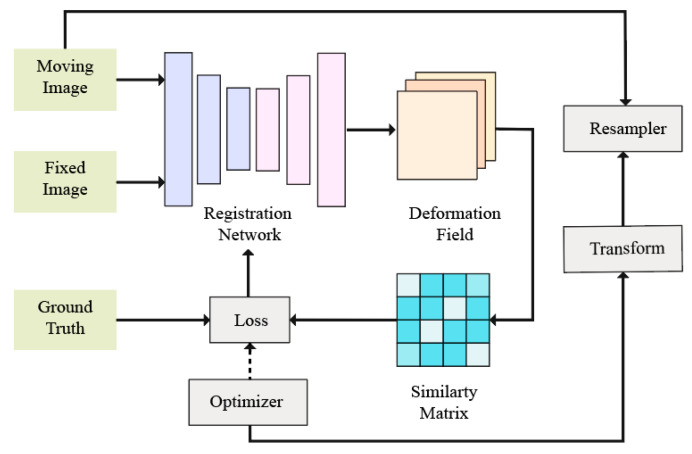
Image registration core framework.

Figure 10 is the core framework of image registration. Usually, image registration mainly goes through the following four steps: (1) feature points are obtained by extracting features from two images; (2) a similarity matrix is constructed by finding matching feature point pairs by similarity measurement; (3) the image space coordinate transformation parameters are determined through the matched feature point pairs, and we find the maximum relevant point according to the optimization criterion and solve the unknown parameters in the transformation model; (4) we perform image registration on the obtained optimal coordinate transformation parameters to realize the inter-image registration match.

This paper lists the outstanding research results in the field of medical image registration in recent years in Table 8.

#### 3.3.4. Other Applications of AI in Medical Imaging

Image synthesis and enhancement: Image synthesis and enhancement between different medical imaging modalities/protocols is a very active area of research in radiation oncology and radiology. However, medical images have their particularities. It is concentrated in the following aspects: (1) Strong privacy, medical data involving patient privacy cannot be exposed, resulting in relatively few medical data sets; (2) complex structure, different from natural images, medical image data are complex and diverse, usually requiring professional knowledge; (3) acquisition is difficult, and specific image acquisition is not feasible, which not only increases the exposure of patients to ionizing radiation but also bears additional labor and costs. Therefore, many researchers have proposed a series of methods for image synthesis and enhancement; the most popular one is based on generative adversarial network (GAN), which aims to facilitate specific clinical workflows by bypassing or replacing specific imaging procedures.

Integrating GAN in a progressive genetic algorithm to rapidly synthesize ischemic heart disease images with comparable quality to real images. Zhou, et al. [110] proposed a diabetic retinopathy generative adversarial network (DR-GAN) to synthesize high-resolution fundus images that can be manipulated with arbitrary grade and lesion information. Huang, et al. [111] proposed a multi-task coherent mode transferable GAN for unsupervised brain MRI synthesis. It effectively alleviates the problems of patient discomfort, expensive costs, and the unavailability of scanners. Impressive results have been achieved in image synthesis and enhancement by creatively applying deep networks in different tasks. One can only expect a further increase in the number of future missions.

Automatic report generation: Radiology reports serve as the primary means of communication between radiologists and referring physicians. However, writing a clear, correct, concise, and complete report is a daunting task for radiologists, especially in developing countries, due to their high workload, short time, and fatigue. According to statistics, about 4% of reports written by radiologists have errors in interpreting observable visual patterns, such as ambiguity of words, double negatives, undefined modifiers, etc. [111]. These all lead to doctors who may be misled when reading the report. Therefore, to solve the above problem, there has been a lot of research into methods of automatically generating reports with the aim of streamlining clinical workflows, thereby reducing time, errors, and costs.

You, et al. [112] proposed a framework for the automatic generation of AlignTransformer reports, which can be used to calibrate transformers that alleviate data bias issues and model very long sequences for generating medical reports. Chen, et al. [113] proposed a cross-modal memory network (CMN) to enhance the encoder-decoder framework for radiology report generation, where the shared memory was designed to record the alignment between images and text, addressing the multimodal mapping of graphics and text and how to use this mapping for better report generation.

Image retrieval: With the exponential increase in medical data, content-based image retrieval (CBIR) has become a research hotspot. It is a technology for knowledge discovery in massive databases. It extracts the features of input images and quickly searches the image database for similar disease histories in the feature space to assist clinical diagnosis. At present, the main difficulty of CBIR is the huge difference in the content characteristics of medical images obtained by different imaging devices, as well as how to effectively extract the features from the images and associate them with meaningful concepts. The main challenge in the development of CBIR methods is to extract efficient feature representations from pixel-level information and associate them with meaningful concepts. The ability of deep CNN models to learn rich features at multiple levels of abstraction has piqued the interest of the CBIR community.

Similar images are extracted by an image feature generation method and network community detection technology, which can be used to extract similar images from large-scale X-ray databases. Fang, et al. [114] proposed a triplet hashing (ATH) network to preserve classification, ROsI, and few-shot information by learning low-dimensional hash codes. This method can effectively guide medical images based on medical records and alleviate the problem of sample imbalance. Gu, et al. [115] proposed a depth map-based multimodal feature embedding (DGMFE) medical image retrieval framework which exploits the visual similarity between images to build a multimodal graph model to explore potential information to achieve image retrieval tasks.

Although there are many examples of research on content-based image retrieval at present, there is a lack of successful applications in practice. Compared with the achievements in other fields, it will take a certain amount of time.

In the future, the diagnosis of artificial-intelligence-assisted medical images will continue to become more automated and user-friendly and can give doctors and patients more and clearer quantitative results, developing in three directions. The first is that image processing technology will be faster and more accurate and can assist doctors in more diverse diagnoses. The combination of quantum computing and AI medical images, the development of hardware, and the open source of data sets of various diseases are all beneficial to the development of MPT and also provide a research basis for the continuous exploration of more types of diseases. Secondly, deep learning and artificial intelligence continue to explore semi/unsupervised methods. The application of low annotation levels reduces labor costs while lowering the threshold for research, attracting more scholars to study medical imaging and continuously improving the performance of non-massive data-driven methods. It can also accurately assist doctors in diagnosis while reducing costs. Finally, it is the combination of image technology and other clinical contexts, as well as the fusion of more data, that allows the results to be transformed from a single image space to patient-level information, combined with pre-diagnostic, intra-diagnostic, and prognostic information, to provide doctors with more comprehensive and accurate information suggestions for reference.

### 3.4. The Application of AI in Electronic Health Records

#### 3.4.1. EHR Data Characteristics

Electronic health records (EHRs) are electronic health and medical data that record medical information such as the patient’s medical history, medication administration records, laboratory results, clinical records, and treatment costs. EHRs intuitively display the results of patient treatment and examination. At the same time, they also imply the causative factors of a certain disease, the side effects of drugs, the implicit relationship between diseases, the correlation information between examination data and diseases [116], etc. The development of machine learning algorithms and big data technology enables EHR data to be used as a source of massive data for building classification or prediction models in AI-assisted medical applications, laying the foundation for building clinical decision support systems and personalized precision medicine. EHR data has gradually become the core of health care research AI-assisted auxiliary medical applications [115,116,117].

(1) Patient representation based on discrete medical concepts involves extracting patient features from discrete concepts such as international classification of diseases (ICD) codes or medical texts. How to realize the relation extraction of high-dimensional medical text data and the alignment of medical codes in different standards is the main difficulty of this type of patient representation.

Reference [45] developed a natural language processing (NLP) framework based on UMLS MetaMap and BioWordVec, which constructs patient representations using discrete EHR data and then applies clustering and association rule mining to discover patient clusters and associations between patient attributes. By applying the framework to breast and colorectal cancer patients, this research finds associations between various symptom types in breast and colorectal cancers. Paper [116] introduces a knowledge graph system for EHR data which transforms EHR data into a semantic patient-centralized information model so as to perform reasoning through semantic rules to identify important clinical findings and neglected clinical information in EHR data. This paper identifies 2774 patients who met the diagnostic criteria but were neglected, effectively exploiting the hidden unused information in the EHR data.

(2) The patient representation based on time series medical data is designed to represent the patient by time series vital signs data such as respiratory rate, heart rate, blood pressure, etc. The difficulty of this type of representation is how to establish a model to automatically extract associations between patient’s signs and disease symptoms in different time series. At the same time, the problems of uneven sampling and asynchronous sampling of time series data should be taken into account.

In [41], a bidirectional deep learning model (BRLTM) is developed to aggregate diagnosis codes, procedure codes, drug codes, and clinical records. Then, it mines the temporal relationship of records to obtain patient representations and predict depression. Reference [42] proposes a prediction model based on the attention mechanism, which learns patient representation by perceiving the contextual information and temporal relationship of disease codes in EHR data, thereby diagnosing diseases in patients. The model proposed in this paper has shown excellent predictive performance with heart failure, diabetes, and chronic kidney disease datasets. In [118], a temporal tree model based on temporal hierarchical representation and temporal co-occurrence is proposed to represent patients, and doc2vec embedding technology is used to enhance the patient representation, thus realizing the computation of patient similarity.

(3) Patient representation based on multimodal data requires the fusion of diagnostic codes, medical texts, vital signs data, medical images, and other data from different modalities to express patient characteristics. The main difficulty associated with it is how to solve the heterogeneity of data and how to obtain the associative learning of EHR data from different modalities.

**Figure 11:**
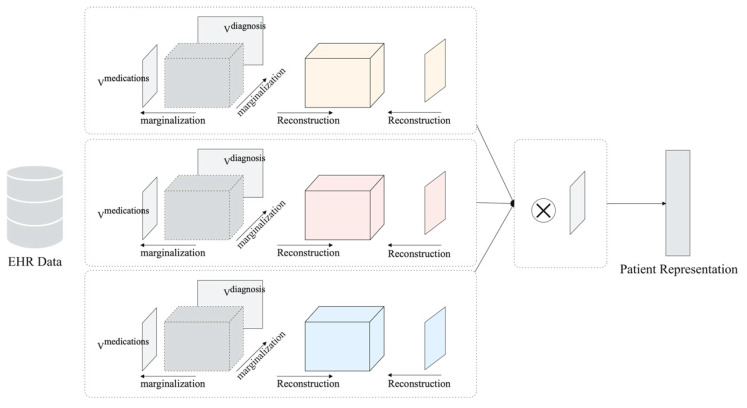
Collective hidden interaction tensor decomposition model [82].

Reference [12] integrates various heterogeneous data, such as diagnostic ICD codes, medication time records, and medical image descriptions to characterize patients. It then uses gradient boosting, multi-layer perceptron and convolutional neural networks-long short-term memory (CNN-LSTM) models to achieve the detection of sepsis. Reference [118] uses a matrix-based representation to perceive the multi-source structure of clinical EHR data, and it uses CNN to extract patient feature representations to identify patients with Kawasaki disease from incomplete data efficiently. In [119], a collective hidden interaction tensor decomposition model (cHITF) is proposed to infer the correspondence between the diagnosis and medication records of multiple different modalities, thereby jointly learning the patient representation. Figure 11 shows the specific framework of cHITF. Experiments on the MIMIC-III dataset show that the patient phenotypes learned by cHITF are more clinically relevant.

Table 9 shows the methods and deficiencies of recent research on EHR data-assisted patient representation learning.

#### 3.4.2. EHR Data-Assisted Disease Diagnosis and Prediction

One of the most extensive applications of EHR data-assisted health care is disease diagnosis and prediction. Using EHR data to diagnose diseases can effectively improve the accuracy of diagnosis and prediction, thereby helping patients achieve disease prevention through early warning, simplifying clinical decision-making, and reducing medical costs. When using EHR data for disease diagnosis and prediction, some studies achieved disease diagnosis and prediction based on patient representation [41]. Other research has tried to directly mine the effective disease features from the EHR data to classify or predict the disease. Such research can still be divided into three types according to the type of EHR data used: disease diagnosis and prediction based on discrete medical concepts, time series medical data, and multimodal data.

(1) Disease diagnosis and prediction based on discrete medical concepts often face the challenge of high dimension and sparsity. Reference [120] proposes a semi-supervised multi-task learning which treats the prediction of the glomerular filtration rate (eGFR) state at a single time point as a task according to eGFR, pathological classification, and other structured EHR data so as to predict the short-term evolution of chronic kidney disease.
(2) In examining other studies of disease diagnosis and prediction based on time series medical data, the literature [121] proposes a model based on third-order tensor decomposition to capture ternary correlations involving additional clinical attributes or temporal features from EHR diagnostic records, thereby predicting the incidence of chronic diseases. Reference [85] uses an attention-based Bi-LSTM model to capture the temporal information of time series EHR data to predict the risk of cardiovascular disease in patients.
(3) When performing disease diagnosis prediction based on multimodal data, typical cases involve the fusion of physiological signal features and EHR structural data and the fusion of medical images and EHR structural data to achieve disease diagnosis or prediction. Advanced signal processing techniques, such as Taut String estimation and dual-tree complex wavelet packet transform, are used to extract features from ECG signals to predict the onset of complications in cardiovascular surgery. In addition, chest X-rays and clinician confidence levels in the diagnosis were used as measures of label uncertainty to make a diagnosis of acute respiratory distress syndrome (ARDS) [122].

#### 3.4.3. Other Applications of EHR Data

A crucial application of EHR data in AI-assisted intelligent medical systems is the hospital resource utilization prediction. Reference [122] uses LSTM and Gate Recurrent Unit (GRU) to capture temporal information between clinical data and utilizes a convolution-based multimodal architecture to extract features from patients’ clinical records. By utilizing multiple word embedding techniques, such as Word2Vec, FastText, and their combination, this reference obtains low-dimensional representations of patients, thereby predicting patient mortality and hospitalization time and managing hospital resources. Reference [44] uses a fuzzy-division-enhanced local weighted decomposition machine to learn the hospitalization characteristics of patients in EHR data and considers the possibility of patient readmission, so as to manage medical resources.

Another application of EHR data is the prediction of patient mortality and clinical endpoints, which helps physicians make decisions to stop treatment at the right time so as to effectively eliminate overtreatment. A bidirectional variational recurrent network randomly repeats the imputation of missing data in the EHR and represents the uncertainty in the distribution of imputed values. Thus, it can utilize the uncertainty to update the hidden state in the GRU unit to realize the prediction of patient mortality. Reference [123] proposes an embedding model based on deep learning which constructs a multi-level corpus for the categorical variables in EHR data. According to the order of cross-domain interaction, it ranks the categorical variables in EHR data, thereby sequentially learning attribute-level representations of cancer patients and predicting the clinical endpoints of cancer patients.

In addition, the EHR data can be used to calculate the similarity between patients and find similar patient clusters. Reference [118] obtains patient representations by using the time tree model and doc2vec embedding technology, thereby realizing the calculation of patient similarity. Reference [124] develops a probabilistic model based on Gaussian mixture models to find patients with specific clinical characteristics and uses hierarchical clustering, supported by Kullback–Leibler divergence, to create a robust low-dimensional space so as to identify groups of patients with similar health conditions. Similar or identical clinical treatment regimens can be used for patients in similar or the same cluster.

#### 3.4.4. Challenges Faced by EHR Data-Assisted Health Care

**Figure 12:**
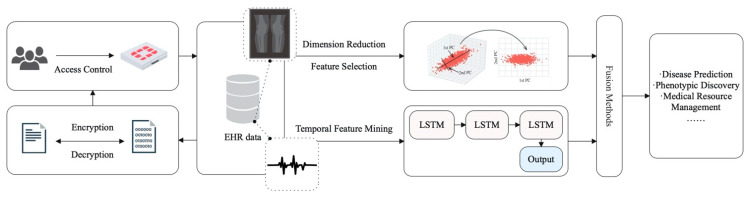
Challenges faced by EHR data-assisted health care and common solutions.

There are already many examples of research and applications of EHR data-assisted health care in academia and industry; however, when using EHR data to assist medical treatment, researchers often face the challenges of privacy, heterogeneity, time series, high dimension, and sparsity. EHR data contains a large amount of patients’ privacy information and covers health data from different sources, different types, and different modalities. An inpatient’s EHR data may record structured diagnostic codes, time series vital sign data such as blood pressure and heartbeat, as well as unstructured medical text data such as diagnostic analysis, clinical description, and unstructured medical imaging data such as CT and MRI. At the same time, patients usually do not complete all examinations and treatment activities, so EHR data shows typical high-dimensional features. Figure 12 shows common solutions in research.

In terms of privacy challenges, techniques such as access control, security authentication, and encryption are used to protect patients’ private information.

The challenge of heterogeneity is often solved by fusion methods. At present, most research focuses on using deep learning and joint learning frameworks to fuse data from two different sources and different structures [119,120,121].

The time series challenge of EHR data requires mining the temporal association of data. Current research usually uses time series models such as exponential smoothing models or deep learning algorithms such as LSTM networks [41].

When dealing with the high-dimensional challenge, appropriate dimensionality reduction and feature selection are required. When performing dimensionality reduction and feature selection, many studies choose methods of clustering [80], tensor decomposition [82], or support vector machines.

At the same time, there are still two major problems to be solved in the research of EHR-assisted health care: (1) With the continuous enrichment of codes, texts, the time series of vital signs and medical images in EHRs, we need to establish how to realize clinical decision-making and automatic reasoning from the massive and complicated EHR data; (2) we also need to establish how to improve the interpretability of the model while improving the performance of the model and provide explanations that doctors can understand and agree with, thereby promoting the clinical application of EHR-assisted health care. Therefore, EHR-assisted health care urgently needs more exploration.

### 3.5. The Application of AI in Health Management

**Figure 13:**
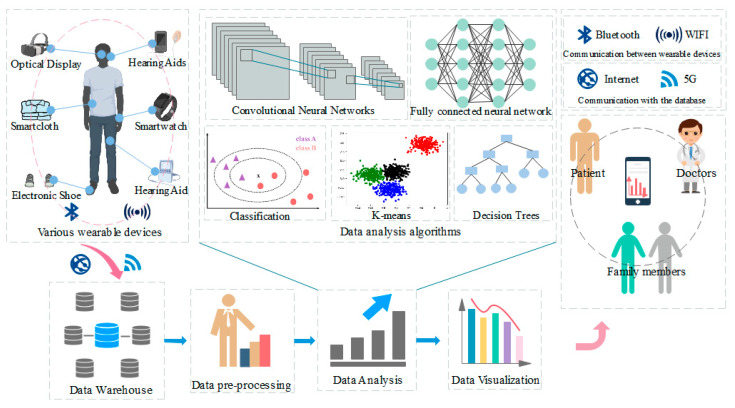
Framework diagram of health management system.

With the support of 5G communication technology, smart IoT terminals are gradually becoming a part of the medical field. Smart wearable devices can measure blood pressure, heart rate, and blood oxygen, and a variety of wearable devices can monitor the user’s physical condition in real-time. In health management, wearable devices can collect real-time monitoring data from users and then transfer the data to the backend for storage. After pre-processing the raw data, the health management system can use AI methods to analyze and mine the data and generate analysis reports. Through electronic reports and data visualization, the analysis results are fed back to doctors and users, and assist doctors in mastering the users’ physical conditions, as shown in the framework in Figure 13. The health management system can monitor users’ physical condition in real-time and does not require doctors to communicate with users face-to-face, which not only spreads medical resources to remote areas but also saves time for users in terms of seeking medical treatment. In the context of the global outbreak of COVID-19, telemedicine through wearable devices can solve the problem of patients’ access to medical care. However, the process of data collection and transmission involves the possibility of data leakage, which poses a threat to users’ privacy. In this section, the various aspects of health management, including wireless mobile treatment, medical data fusion and analytics, medical data privacy protection, and health management platforms, will be introduced specifically.

#### 3.5.1. Wireless Mobile Treatment

In life, wireless devices are closely connected to us, such as smart bracelets, wristbands, subcutaneous sensors, etc., which can be placed on specific parts of the body for the data monitoring of different performance indicators. Users can wear different wearable devices as needed to manage diseases such as diabetes and epilepsy [125] in addition to monitoring their physical condition, and such devices can even determine whether the user is infected with COVID-19 [126] through each data indicator. Wireless devices generate monitoring data every moment and transmit data such as blood pressure, temperature, and oxygen saturation to the background through communication methods such as Bluetooth and WIFI, which can be used by researchers for data analysis.

Wearable devices exist in various forms. Textile-based wearable devices, with sensors implanted in clothing and placed at the user’s waist, can track heart rate changes for monitoring and analyzing cardiovascular diseases. Detection of the user’s body temperature can also be achieved with a sensor close to the skin. Biofluid-based wearable devices can perform disease surveillance by detecting the composition of important secretions such as sweat and tears produced by the body. A wearable device for colorimetric sensing of sweat has been developed in the paper for the detection of chemical composition [127]. The wearable device can also be used for timely drug control and delivery for disease treatment based on the measurement of body indicators such as body temperature, heart rate, sweat, and so on. For example, timely drug delivery to the eye through the control of the system is used for the treatment of glaucoma.

Among various kinds of wearable devices, in addition to single monitoring of health indicators, they can also integrate various monitoring data to complete the monitoring of specific diseases, monitor patients’ physical conditions in real-time, and guarantee life safety. By tracking the wearable devices worn by pre-diabetic patients, the patients’ activities, and combining these elements with medical history information, it is possible to accurately determine the changes in blood sugar control of patients, which is convenient for patients in regard to controlling blood sugar and preventing further deterioration of the disease. The paper [88] combined implantable EEG electrodes and a wearable device for seizure detection and used machine learning algorithms to analyze patient data to assist in the diagnosis of epilepsy and change the management of epileptic patients. The paper [126] proposes a decision tree algorithm. It uses data from smart bracelets, combined with characteristics such as ambient temperature and own temperature changes, to predict whether a person is infected with COVID-19. Heart rate changes before and after COVID-19 infection were then analyzed.

Among all diseases, the management of chronic diseases such as diabetes and cardiac arrhythmias has been a major challenge. This requires not only constant monitoring of the body but also timely management of changes that occur in the body. If the patient frequently goes to the hospital for observation, it will consume a lot of time and energy. With the help of wearable devices, integrated smartphones can then enable atrial fibrillation [46] and the implementation monitoring of cardiovascular diseases [47]. The wearable devices in different parts of the body are interconnected by short-range communication such as Bluetooth, NFC, and WIFI, and the results are synchronized to the smartphone for mobile medical management. Doctors can access the data in real time to make a diagnosis of the patient’s physical condition, and family members can also access information about the patient’s physical condition through the management platform. For patients suffering from cardiac arrhythmia, real-time monitoring of the physical condition is required to prevent unexpected conditions. The paper [46] describes mobile health technologies for arrhythmias, including ECG and non-ECG based modalities with detection devices for commercial use, such as smart detection bracelets. The paper [47] proposed a wearable device-based cardiovascular disease monitoring system for patients with COVID-19 which not only accurately predicts the cardiovascular health of patients but also effectively reduces the mortality of COVID-19.

In the context of the global outbreak of COVID-19, it has become difficult for patients to travel to hospitals for medical treatment. Telemedicine can go some way toward meeting patients’ needs when they are unable to travel to a hospital to meet with a doctor face-to-face. For non-emergency patients, telemedicine can be more convenient. In areas where medical resources are not evenly distributed, telemedicine can also allow people in remote areas to enjoy quality medical resources and save a lot of time and costs through online visits [127].

Through a variety of telemedicine platforms, patients can manage their diseases. The physician acts as a teacher in the remote platform, making judgments based on the patient’s condition and answering questions from the patient. The paper [128] addressed the problem of obesity management in remote areas through a telemedicine platform. The paper [129] outlined telemedicine services for pregnant women, heart failure patients, diabetic patients, and stroke patients, addressing the issue of geographical differences in patients.

#### 3.5.2. Medical Data Fusion and Analytics

In wireless mobile diagnosis and treatment, wearable devices perform data monitoring of various body indicators and transmit the data to the management platform for data analysis. However, due to the wide variety of wearable devices, even from different manufacturers, fusion and pre-processing of the collected patient data is a key step before data mining. The difficulties faced by medical data in fusion and pre-processing include data noise, missing data, and data scarcity. The solution is mainly to solve the problem of small datasets using data augmentation. The paper [48] analyzes textual data to predict patients with poor visual prognosis by fusing patients’ electronic health records and free-text data.

To better serve patients and fully extract valid information from the data, the processed data needs to be analyzed and mined. Currently, most researchers in the field of data analysis and mining choose to use machine learning and deep learning methods, while others use association rules for information extraction from data. The paper [48] reviewed three artificial intelligence algorithms, which are fuzzy logic, genetic algorithm, and hierarchical analysis. Based on these, clustering, classification, and association rule algorithms were investigated to extract useful information from health data. The paper [9] proposed a deep learning-based medical data mining algorithm that discretizes complex medical data, analyzes the direct mapping relationships of the data using convolutional neural networks, and then extracts the association rules between the data.

In the raw data collected through wearable devices, most of them are individual numbers, and the data collected through medical imaging such as CT are black and white two-dimensional images. These raw data are not favorable for doctors in terms of making a diagnosis by direct observation. It would be more intuitive to present these data in the form of graphics or 3D images. Presenting the results of data analysis and mining in the form of an image interface would also allow the patient to clearly see the physical condition and body changes. The paper [130] reviewed the visualization techniques for 3D medical images and summarized the free platforms used for medical image visualization. The paper [94] reviewed the visualization tools in the healthcare industry, summarized the reasons and significance of visualization, and compared the types and advantages of different visualization tools. The paper gives examples related to healthcare data visualization and explains how data visualization can be applied to the healthcare industry.

The application of visualization technology to the medical field can provide a lot of convenience to patients and doctors; however, due to the special nature of medical data, patient privacy has to be protected during the visualization process. The challenges faced in medical data visualization mainly include multiple sources and modalities of medical data, too few public datasets, patient privacy, and ethical level considerations.

#### 3.5.3. Medical Data Privacy Protection

In the health management platform, from data collection to data transmission and finally storing data in the backend server, as well as the subsequent data mining and analysis process, all the steps must perform a good job regarding data confidentiality to prevent data leakage from threatening the privacy and security of patients. Considering the three steps of data transmission, storage, analysis and visualization, all of them can take corresponding technical means to guarantee the security of medical data [131]. The paper [48] reviewed the challenges and solutions faced in the field of medical data protection, including the data security of electronic health records and the data security of electronic case systems, and information encryption algorithms were listed in the paper to address data security threats. The relevant paper on privacy protection was compiled and selected to summarize some of the paper, as shown in Table 10.

#### 3.5.4. Health Management Platform

Whether it is wearable devices for body monitoring and disease management or telemedicine to enable patients to access medical care online, patients need to connect with their doctors through a comprehensive health management platform. As a link between data, doctors and patients, the health management platform not only collects data generated by wearable devices but also synchronizes the results of data analysis and mining in a visualized form to the platform, where doctors and patients can query and understand their health conditions and communicate with each other in a timely manner.

A complete health management platform for a certain disease should contain data collection, data storage, data analysis, and data result in presentation. The paper [52] proposed an open-source software architecture describing a health management platform that includes data collection, data transmission, data analysis processing, and data visualization. The platform collects data from wearable devices and uses encryption technology to transfer the data to the backend storage. It also combines historical data to correlate them with specific diseases, based on which the data are analyzed and mined by machine learning methods, and the analysis results are then fed back to users through the platform. The paper [135] constructs a diabetes health management platform based on GCM where patients monitor blood glucose changes in real-time by wearing blood glucose monitoring devices such as GCM, transmit the collected data to the platform for analysis, and provide advice to patients on diet and medication based on the data results.

In the context of the global epidemic of COVID-19, online access to medical care becomes a better solution. During home isolation, health testing is still possible through IoT devices and communication with physicians is possible through a home health management platform. The paper [136] designed a doctor–patient management platform that monitors basic patient data through medical collection devices, transfers the data to a data warehouse for storage after data collection, and updates them quickly to solve the problem of incomplete patient data and to bring hospitals, doctors, and patients together.

With the development of society, it has become a trend for human beings to live in communities. It has also become a norm for the elderly to age in the community. A Colombian healthcare facility’s digital health platform integrates operational, clinical, and business data repositories with advanced analytics to improve decision-making for population health management. The paper [137] proposes a community-based aged care model that connects children, the community, and emergency care by collecting health information from older adults to build a network of good relationships.

In the future development of society, as people pay more and more attention to physical health, coupled with the serious aging of the population, the use of wearable devices for health management and elderly services will become a major direction of future development. Using AI intelligence to assist health management not only monitors users’ physical condition in real-time but also helps doctors to obtain patient information in an timely manner. The emergence of telemedicine enables people in remote areas to enjoy quality medical resources, and community management platforms are more adaptable to the new elderly care model. However, in the development of health management, many links are involved, and many difficulties will be faced in addition to the challenges of developing and communicating wearable devices, as well as the difficulties of data storage, pre-processing and mining, the rational use of visualization tools to display the analysis results, and, more importantly, the protection of data security and users’ privacy and the challenges of information security [51].

### 3.6. The Application of AI in Medical Robots

Medical robots can be traced back to the first neurosurgical brain biopsy performed in 1985, and in the three decades since then, medical robots have been developed for laparoscopic surgery, prostate biopsy, ophthalmic surgery, and other medical fields, and surgical robots, such as Da Vinci, Verb, and MAKO, have emerged that are widely used in the world [138]. Medical robots integrate equipment tools such as miniature cameras, surgical sutures, and robotic arms, and they have the advantages of high accuracy, flexibility, and control. They have largely solved the shortcomings of traditional surgery performed manually through machine automation for precise positioning and diagnosis, remote treatment, and high-quality patient care, and have significantly reduced the workload required by healthcare professionals to diagnose and treat patients.

Artificial intelligence robots are assisting doctors in completing complex tasks from positioning and diagnosis to surgery, making medical treatment more intelligent and scientific, thereby providing more convenience to humans [139]. According to the application level from shallow to deep, research in recent years can be divided into three major categories: surgical robots, rehabilitation robots and intelligent assistive robots, as shown in Table 11.

Because surgical robots have precise positioning and do not produce physiological tremors and fatigue, they can help achieve smaller surgical trauma and reduce postoperative complications. Their application range is very wide and includes orthopedics, heart treatment, bowel treatment, gynecology, and neurosurgery. Examples include the swan for joint replacement; Tumai, China’s first comprehensive coverage of the chest, abdomen and pelvis; and the Mona Lisa for prostate puncture. The path correction method proposed by S. Lu. et al. can guide and correct the movement trajectory of the needle to the original correct planned path by partially re-planning the robot’s motion; and the cable-driven soft tissue surgical robot da Vinci and Raven [140]. However, existing surgical robots are expensive, bulky, require human supervision, and have not yet achieved complete autonomy.

Surgical robots have not solved surgical prognosis issues such as rehabilitation training. The emergence of rehabilitation robots provides convenience for helping patients carry out rehabilitation training. This involves conducting long-term and effective repetitive training of patients’ damaged limbs through limb tracking, motion trajectory prediction, guidance and driving to achieve the purpose of treatment. Miguel Nicolelis and others from the United States used three major training technologies [141], brain-computer interface, lower limb exoskeleton rehabilitation robots, and virtual reality, to achieve significant recovery of spinal cord injury patients from a state of complete paralysis to lower limb muscle function and sensory function. Mehrabi and others designed a lightweight, safe, friction-driven dual-degree-of-freedom ankle rehabilitation robot that can be installed on any mobile platform to guide and help patients perform ankle rehabilitation training; the portable planar passive rehabilitation robot PaRRo can complete the patient’s arm of rehabilitation treatment [142]. Unfortunately, rehabilitation robots have privacy and security issues in regard to patient records, genetics, and other data.

For work such as disease diagnosis and remote assistance, auxiliary robots can achieve auxiliary diagnosis through visualization, high precision, and real-time performance, thereby greatly reducing the repetitive workload of manual positioning and classification. For example, the blood collection accuracy of China’s first blood collection robot reaches 92%, which is basically the same as the accuracy of blood collection by medical staff [143]. However. The interpretability of potential artificial intelligence models is low, and there are potential risks when robots assist in diagnosis and treatment, so their results can only be used as auxiliary reference for doctors. In addition, both patients and doctors are inherently skeptical about artificial intelligence diagnosis and treatment technology.

**Figure 14:**
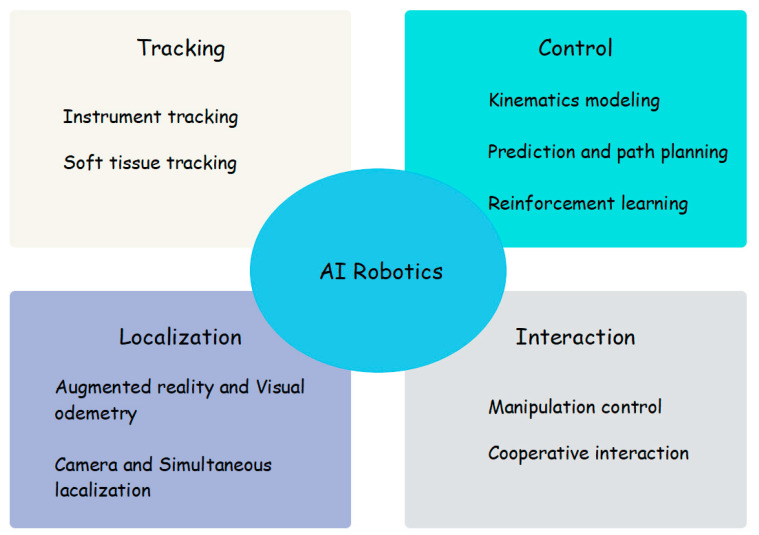
Artificial intelligence technology applied to AI robotics systems.

Deep learning plays an increasingly important role in the development of robots. Object detection, tracking, and image segmentation of surgical targets are important aspects of surgical robots performing surgical procedures. The combination of deep learning algorithms for computer vision, pattern recognition and real-time decision-making with clinical medicine enables AI robots to perform well in pre-operative planning and intra-operative guidance, and they can complete medical-related tasks with high accuracy and safety [143]. Deep convolutional neural network models such as AlexNet, ResNet, and DenseNet make feature patterns easier to understand and image classification more accurate. As shown in Figure 14, this figure outlines the more popular artificial intelligence technologies applied to AI robot systems in recent years from four aspects: target tracking, positioning, control, and human–computer interaction.

In terms of tracking and positioning, deep learning algorithms break through the traditional method of object tracking through local optimization. Existing deep neural network algorithms combined with micro cameras embedded in robots can quickly and accurately detect and locate moving equipment and tools, thereby effectively detecting, locating, and segmenting target objects in complex images, such as U-net and Attention. Sarikaya et al. [144] used a weak supervision method to automatically generate binary annotations to mark the tool space information in the data set and completed real-time tracking of surgical instruments through LSTM and CNN technology. This method effectively improves the accuracy of surgery. Aviles et al. [145] proposed a neural network based on the LSTM-RNN structure, which learns and extracts visual geometric features through supervised learning methods to accomplish accurate estimation of force magnitude. Experimental results show that this method can achieve a recall rate of 0.98 and an average root mean square error of only 0.02 N.

In terms of control and interaction, algorithms such as deep convolutional neural network (DCNN) and recurrent neural network (RNN) are used to extract data for nonlinear kinematic modeling, thereby achieving auxiliary segmentation and recognition of complex surgical activities. Deep learning models such as multi-scale LSTM (MS-LSTM) Dueling [54] are used to predict multi-joint motion trajectories and reduce motion errors between the user’s arm and the robot arm, thereby effectively guiding patients to perform bilateral rehabilitation training. Technologies such as motion planning, control, and perception can be used to plan robot arm movements to complete automatic correction in surgical paths. Models such as reinforcement learning are used for analytical modeling to efficiently demonstrate complex operations such as automatic suturing of soft tissue and high-precision spinal needle injection. For example, B Thananjeyan et al. [55] used the Deep RL method to learn tensioning strategies in a simulated environment of tissue cutting or removal, effectively learning the patterns of surgical scissors and tissue. E. De Momi [146] et al. used an artificial neural network architecture to effectively accomplish human–robot control of planned surgical motion trajectories. Oyedotun et al. [60] used convolutional neural networks and stacked denoising self-encoders (SDAE), and they were able to perform complex gesture classification tasks with high accuracy, effectively improving the safety and reliability provided by robotic gesture control.

Although medical robotics has only emerged and developed in the last three decades, its applications in clinical medicine and other fields have had a significant impact on the entire healthcare industry along with the robotics industry. AI robots combined with machine learning related algorithms can complete target tracking, system modeling, real-time decision-making, and control more efficiently and quickly. Robotic systems can assist medical staff in precise diagnosis, minimally invasive surgery, rehabilitation care and other processes for patients through automated or semi-automated methods, greatly reducing the work intensity of medical staff. In the future, artificial intelligence technology and robot systems will be more closely integrated, and medical robots will become intelligent, scientific, and rational [147]. For example, we will develop artificial intelligence algorithms that are easier to explain and improve the algorithm’s decision-making capabilities; use reinforcement learning and other methods to improve the autonomy of AI robots in performing complex tasks and reduce surgical risks and uncertainties; try to develop more comprehensive functions as well as light-weight and cheaper robotic systems and more complete laws; develop more personalized 3D bioprinted organs; utilize autonomous learning of complex tasks; and optimize non-invasive surgery. The treatment model of AI robots may also undergo profound changes.

## 4. Opportunities and Challenges for the Development of Artificial Intelligence in the Field of Medicine

In recent years, with the continuous development of artificial intelligence technology, smart medical treatment has become a hot spot in the medical field. In developing countries with a large population, the degree of population aging is deepening, the incidence of common diseases, such as chronic diseases in the elderly, is increasing year by year, and the dilemma of insufficient medical resource allocation is becoming increasingly serious. In China, case experts conducted a statistical analysis of hundreds of thousands of cases and found that the country’s average misdiagnosis rate was 33 percent, while the rate of outpatient misdiagnosis was as high as 50 percent. Artificial intelligence can make medical diagnoses quickly and accurately and help doctors make better decisions in terms of medicine. Health care is one of the fields with the greatest potential for AI application, which has been recognized in the medical industry. As a highly comprehensive interdisciplinary technology, AI will find more and more applications in the medical industry. Therefore, for the future development of artificial intelligence in the medical field, it is very necessary to study the opportunities and challenges it may face.

### 4.1. Opportunities for AI-Assisted Healthcare

(1) Breakthroughs in AI technology

As early as 1980, some scholars started preliminary attempts to combine artificial intelligence with medicine. Subsequently, many researchers engaged in this field one after another and conducted a lot of exploratory work on the application of artificial intelligence in TCM, with commendable constructive achievements [148]. However, these research results did not meet people’s expected standards and were always at the theoretical level; therefore, they could not be used in practical medical diagnoses. In recent years, the breakthrough of computer hardware computing ability and the massive data generated by human beings on the Internet have provided a physical basis for the computation of artificial intelligence [149,150,151]. Artificial intelligence technology has developed in a gush-like style after researchers put in a lot of research work.

In addition to hardware advances, there are also improvements in convolutional neural network models (CNN) and parameter training techniques. In 2012, Hinton’s students used ImageNet to significantly reduce the error rate on ImageNet, beating Google’s current state-of-the-art image classification algorithm, which has attracted a large amount of research and development in deep learning in the industry [152]. Moreover, Werbos reinvented BP and introduced it into ANNs, further improving the application scope and accuracy of neural networks. Today, deep learning networks are popping up and getting better at tasks, surpassing humans in areas such as speech recognition, image recognition, and video games [153]. AI has come close to, and even exceeded, the accuracy of CT-assisted diagnosis by imaging doctors [154]. At the same time, deep learning is also advancing towards the direction of deeper network layers and more complex and efficient structures, and the research and development of GPU is also continuously and rapidly advancing for the training of increasingly complex AI models [155]. As computing speeds up and data on the web becomes richer, new AI technologies are constantly being developed, raising hopes for the future.

(2) Supported by public health data

The core of artificial intelligence involves algorithms, and the basic condition involves computing power and data. In terms of development, any intelligent technology needs to acquire information from massive data for continuous learning and to make fuzzy judgment on new data in terms of decision-making according to the rules contained in existing data. In the medical field, the effectiveness of big medical data is the basis of smart medical applications. With the popularization of the Internet, many medical institutions, administrative institutions, and health management institutions have begun to use the Internet, which also provides conditions for the acquisition of medical big data [156]. In addition, many scientists and research institutes are working to collect and share more data to expand the medical public data set. Through systematic data integration, Peking Union Medical College Hospital standardized collection and management of clinical information and built a new medical platform of intelligent scientific research ecology and a specialized disease database.

Existing medical imaging technologies provide researchers with massive data for numerical analysis of various methods, and some large auxiliary medical systems have used over 2.3 million X-ray images to enhance algorithms [29]. In developing countries with large populations, the volume of data has a great advantage, which can be used to train and improve the performance of artificial intelligence models. Moreover, in recent years, medical data has continued to be structured and standardized, and the sharing degree of medical information has also gradually improved, further promoting the application of artificial intelligence technology in the medical field [157].

(3) Commercial market push

Smart medicine is still in a period of preliminary exploration, and the application of artificial intelligence in the field of medical and health has been favored by capital. In just three months in 2017, more than 30 companies in the smart healthcare business were funded, and HC3I recently released a report showing that AI healthcare applications are expected to reach USD 25.4 billion by 2035. As a result, increasingly traditional companies and Internet enterprises attract IT talents from colleges and universities to make breakthroughs in deep learning technology and provide users with more intelligent medical products and services. Big tech companies such as IBM, Google, and Microsoft are all scrambling to grab market shares in smart healthcare, predicting that AI could explode in the medical sector in the future and divide the market more carefully [158].

(4) The impact of artificial intelligence in the COVID-19 pandemic

Since 2019, people around the world have been adversely affected by the novel coronavirus disease (COVID-19) pandemic. As of now, the COVID-19 pandemic has lasted for more than two years, causing over 500 million infections and more than 6 million deaths. AI and deep learning are playing a crucial role in diagnosing, treating, and preventing many diseases during this epidemic.

In addition to pandemic workers and biological scientists, computer scientists are also working on the response to this devastating pandemic, helping to combat the disease and improve health care facilities. Because COVID-19 is highly contagious and latent in people and can affect large numbers of people in a short period of time, it has resulted in overcrowded hospitals and inadequate medical workforces and equipment. To prevent the above situation, K. K. Srinivas et al. [1] proposed a deep learning model named “FPASSA-ANFIS” which can predict the number of confirmed cases in the subsequent 10 days based on historical data and calculate the quality of each solution through fitness function. Solutions with a high error rate will be discarded. Solutions with a lower error rate will be selected to provide a reliable basis for local governments to adjust epidemic prevention policies.

Because COVID-19 is a form of pneumonia, doctors need to distinguish it from other types of pneumonia flu in their diagnosis. However, while people might take time to distinguish the differences, AI can achieve this easily. S. Degadwala et al. and K. K. Srinivas [62] improved the convolutional neural network model to classify novel coronavirus infected persons and other non-infected persons. With AI-assisted diagnoses, potential cases can be found early, minimizing the speed of transmission and saving more lives. Vaccination is the most effective way to prevent outbreaks. In vaccine development, too much time is spent conducting experiments. Machine learning can be used to speed up the process, providing insights to immunologists by finding the most useful models. B. N. Adday et al. [63] found that, by implementing this method, multiple antibodies can be quickly found to prevent the invasion of COVID-19 into the human body and candidate proteins can be developed to predict the vaccine.

In addition, new technologies, such as temperature patrol robots, AI automatic temperature measurement + portrait recognition, and AI evaluation of the effectiveness of drugs, have also played an important role in epidemic prevention and control [159]. During the most severe period of epidemic prevention and control, e-commerce activities supported by artificial intelligence technology became an important pillar in maintaining the normal operation of the social economy. It is certain that, in the process of accelerating the establishment of an economic and social order compatible with epidemic prevention and control, artificial intelligence technology will be increasingly more widely used, and it will also usher in new development opportunities while assuming important missions. At the same time, although the application prospect of smart medical treatment is broad, we still need to face and solve various challenges from various aspects in the future to realize the product’s full potential.

### 4.2. Challenges of AI-Assisted Healthcare

(1) Market fragmentation

In the medical field, although artificial intelligence has broad application prospects, it still faces some challenges. Under the favorable background of the development of artificial intelligence, many enterprises have joined this industry for business expansion [160]. But, in addition to a certain technology, there is a certain degree of competition between enterprises in terms of market shares. Large companies tend to build their own ecosystems and conduct their own research using the vast resources and technologies they already possess. The market has not standardized this emerging field, which will easily lead to a monopoly of one enterprise in this field, inhibiting the innovation and development of other competitors. The lack of communication and collision of thinking between enterprises further leads to fragmentation and hinders the development of artificial intelligence in the medical field.

(2) Traditional thinking and ethics

At present, in medical diagnosis, artificial intelligence technology can only be used as a tool to provide an auxiliary diagnosis basis for doctors and cannot completely replace doctors. In the traditional medical industry, patients have always accepted the diagnosis and treatment modes of looking, smelling, asking, and cutting. Although new medical devices are being used every year to make the diagnosis and treatment of diseases more efficient, patients and doctors alike are instinctively skeptical of artificial intelligence. In addition, artificial intelligence also involves ethical, philosophical, moral, and other issues, which have long been controversial [161]. To overcome the traditional concept of humanistic ethics and be accepted and recognized by the public, intelligent medicine still has many challenging problems to solve.

(3) Limitations of AI technology

Nowadays, the application of AI in many fields has made breakthrough progress. However, many enterprises blindly follow the flow into this cross-industry field due to the policy bonus without considering whether they have enough technical ability to deal with difficulties. AI models with excellent performance face problems such as difficult training, difficult data collection, and high requirements for hardware equipment. Products and technologies in the current market need to be improved in terms of independent research and development ability. For the lack of medical data security protection measures, there are still some technical bottlenecks in the interdisciplinary joint diagnosis algorithm [162].

In addition, when the AI algorithm makes medical decisions in the next step, it may make wrong predictions involving as AI robot accidents, incorrect drug configuration, misdiagnosis, or diagnostic failure, which will threaten the life safety of patients. From 2011 to 2021, more than 400 reports were submitted to the FDA’s Facility Experience Database with the keyword “robot malfunctions and deaths.” The robots in these reports are not necessarily used in the medical field but suggest that AI robots will need to undergo more rigorous testing and evaluation if they are to be used for therapeutic purposes in medicine.

(4) Data Sharing Issues

High-quality medical data sets are crucial for improving the performance of AI applications in healthcare [163]. Although many countries have huge amounts of medical data, most of it is unstructured and lacks a unified standard format, which makes it difficult to fully exploit the true value of the data. For some rare diseases, only a few case samples of data can be collected, which cannot provide enough information for the AI model to learn. However, the model needs to use large-scale data in the training process, and slight data errors may affect the final AI model, which may cause serious negative impacts on patients when applied to actual medical treatment. However, there is no unified standard and standard process for the use of large-scale medical data at present, and the government has not issued relevant laws to give clear instructions on the privacy protection and responsibility norms of AI data [164]. Therefore, the use of medical data in artificial intelligence will inevitably lead to the risk of disclosure of patient information, which will adversely affect personal privacy.

The medical industry itself is an important field directly related to the safety of people’s lives. Every sample of data from patients involves their absolute privacy, which requires us to strictly supervise the entire process of sharing and using medical data. We need to undertake a comprehensive consideration in terms of ethical, legal, security, and other aspects and formulate strict and perfect rules and regulations for the use of smart medical data sharing as soon as possible.

(5) Shortage of professionals

At present, there is a shortage of interdisciplinary talents at the intersection of artificial intelligence and medical care, especially senior talents with more than 10 years of experience. With the lack of such talents, on the one hand, enterprises cannot fully combine the actual medical scenarios to develop AI technology. On the other hand, without such professionals to popularize knowledge for medical staff, it is difficult to prove the effective value of AI in the medical field, which will lead to insufficient acceptance of AI by medical staff or even resistance to AI due to traditional thinking [165].

The application of smart medicine requires professional training for medical personnel. Under this demand, it is particularly important for the medical industry, enterprises, and universities to jointly establish a sound talent training and introduction mechanism. At present, researchers engaged in the intersection of artificial intelligence and the medical field are very scarce. The lack of such talents will make the implementation of artificial intelligence in the medical field difficult.

## 5. Conclusions and Outlook

### 5.1. Summary

In conclusion, AI has numerous applications in the field of medical assistance and is playing an increasingly important role. This study outlines the development history of AI (particularly its development and current status in medical assistance), provides a detailed overview of AI technologies used in the medical field, summarizes the research achievements of artificial intelligence in medicine in six major areas (including genomics, drug development, medical imaging, electronic health records, health management, and medical robots), and analyzes the challenges and opportunities facing AI in medical assistance. A summary of all the literature in this study is shown in Table 12.

Although AI technology still faces different challenges in the medical field, it also contains huge development opportunities. Artificial intelligence provides auxiliary services for human. It can only support doctors, not replace them. At this stage, artificial intelligence is only a basic, repetitive, and replaceable technical service, mainly aimed at laboratories and their scientific research activities.

### 5.2. Future Outlook

Although AI has made significant progress in applications such as medical image analysis, clinical decision support systems, electronic health records, drug development, genomics, and chemical bioinformatics, this is just the beginning of AI’s journey in the healthcare field.

On the one hand, medical issues often involve high complexity and uncertainty, and AI’s machine learning algorithms can handle these complex problems, providing more accurate diagnoses and treatment plans. For example, in drug development, researchers can use AI to analyze large amounts of drug data and bioinformatics data to predict a drug’s efficacy and side effects, facilitating drug development [168]. In gene editing, AI can optimize gene editing technology, improving the accuracy and efficiency of gene editing [166]; even in medical equipment maintenance, AI can monitor and maintain medical equipment, predicting equipment failures, and reducing maintenance costs [167]. On the other hand, the healthcare industry possesses massive amounts of data, and to achieve the analysis and processing of these large datasets, AI researchers will have even deeper and broader explorations of the healthcare field. For example, in health monitoring and telemedicine, by combining IoT, sensor technology, and artificial intelligence, researchers can design health monitoring devices and telemedicine systems, achieving real-time monitoring and remote health management for patients [170]; in medical knowledge graphs and natural language processing, researchers can develop knowledge graph construction algorithms and medical text mining techniques, realizing the automatic extraction, integration, and application of medical knowledge [169]. Additionally, in virtual reality medicine, researchers can build virtual reality technologies, providing virtual medical services and health management, expanding the coverage and scope of medical services [171].

Artificial intelligence data inference is stronger than human data management mechanisms. With the advent of the big data and big model era, unintentional data collection may lead to overly high data accuracy, resulting in unintended data breaches and omissions. Furthermore, the data extrapolation produced by the deep fusion of artificial intelligence and medicine may also extrapolate some data information that should not be known. Therefore, security considerations are necessary in any artificial intelligence and machine learning research, and data security will be a hot research topic.

The development of technology and people’s continuous in-depth research and exploration have driven artificial intelligence to innovate and break through. AI will ultimately achieve the opening up of unknown medical fields, from laboratory to industrial practice, realizing true implementation. The combination of healthcare and artificial intelligence will have a profound impact on human life, improving human health levels and quality of life and even changing human lifestyles.

## Figures and Tables

**Table 1:** Some common AI algorithms.

Algorithm	Property	Description	Advantage	Limitation
Linear regression	Supervised learning	Model the relationship between independent and dependent variables.	1. Easy to implement. 2. Good interpretability, is conducive to decision analysis.	Unable to handle highly complex/non-linear data.
Naive bayes	Supervised learning	Based on Bayes’ theorem and features independence, it uses knowledge of probability and statistics to classify.	1. Robust, easy to implement, and interpretability. 2. Can incremental training.	The data independence is too strict.
K-nearest neighbor (KNN)	Unsupervised learning	Find the K nearest nodes in the high-dimensional feature space.	1. Easy to implement, can incremental training. 2. Can be used for classification or regression tasks; 3. Not sensitive to outliers.	1. high computational complexity. 2. not suitable for data imbalance tasks. 3. Need enough nodes.
Decision tree	Supervised learning	Build probability functions and tree structures to achieve layer-by-layer prediction.	1. Strong interpretability. 2. Numerical and Boolean data can be handled.	1. Easy to overfit. 2. Ignore associations between data.
Clustering	Unsupervised learning	Based on similarity, maximize the distance between clusters and reduce the distance within clusters.	Can handle complex high-dimensional data.	1. Cannot perform incremental training. 2. Influential hyper parameters, it is bad for training.
Support vector machines	Supervised learning	Set the maximum margin hyperplane as the decision boundary (nonlinear data can be processed by kernel methods).	1. Can handle high-dimensional data. 2. Strong generalization. 3. Can solve the small samples problem.	1. Difficult to find a suitable kernel function. 2. Sensitive to missing data. 3. Weak interpretability
Principal component analysis	Unsupervised learning	Use fewer features to reflect the original feature space to achieve dimensionality reduction.	1. Reduce data complexity. 2. Can de-noise to a certain extent. 3. No hyper parameter limit.	1. In the case of non-Gaussian distributions, the results may not be optimal. 2. Cannot handle irregular data.
Artificial neural networks	All seven categories	Connect a large number of nodes to each other according to different connection methods.	1. Can self-learning and generalization. 2. High accuracy.	1. Not interpretability. 2. Huge computational complexity, need sufficient hardware support.
Multi-layer perceptron (MLP)	Supervised learning	A type of artificial neural network model.	1. Universal approximation. 2. High fault tolerance. 3. Able to learn complex relationships; 4.can quickly calculate large-scale data.	1. Easy to overfitting. 2. Requires a large; amount of training data. 3. Low interpretability.
Conditional Random Field (CRF)	Supervised learning	Probabilistic graphical models are used for modeling and predicting sequential data.	1. Handle sequence data. 2. Capable of capturing long-term dependencies in sequence data. 3. Flexible model structure.	1. High computational complexity when dealing with long sequential data. 2. High difficulty in parameter adjustment. 3. Poor interpretability.
Convolutional Neural Network (CNN)	Supervised learning	A neural network consisting of multiple convolutional, pooling and fully connected layers.	1. No need to manually design features. 2. Weights can be shared.	1. Higher computational complexity. 2. easy to overfitting. 3. Poor interpretability; 4. prone to overfitting
Generative Adversarial Network (GAN)	Unsupervised learning	A model consisting of a generator and a discriminator.	1. Highly readable and understandable. 2. Does not require labeled data. 3. Highly flexible: can be used for various types of data and tasks.	1. The training process is more difficult and requires tuning of multiple hyper parameters. 2. Instability. 3. Evaluation is more difficult. 4. Poor interpretability.
Deep Belief Network (DBN)	Unsupervised learning	Models consisting of multiple Restricted Boltzmann Machines.	1. Can learn the distribution of complex data and multi-layer feature representation. 2. Can generate new data samples. 3. High flexibility.	1. Higher computational complexity. 2. Difficult training process. 3. Poor model interpretability. 4. Easy to overfitting.
Gradient Boosting	Supervised learning	An ensemble learning algorithm that improves prediction accuracy by combining multiple weak learners.	1. High accuracy. 2. Higher flexibility. 3. Higher robustness. 4. Decision-making process relatively easy to explain and understand.	1. The process of tuning parameter is more difficult. 2. Higher computational complexity. 3. Easy to overfitting. 4. Need to choose a suitable weak learner.
Boosting	Supervised learning	An integrated learning method for combining weak classifications into one strong classifier through training.	1. Reduce the risk of overfitting. 2. Applicable to various types of data.	1. Easily affected by outliers; 2. Complicated adjustment. 3. High computational complexity.
Random trees	Supervised learning	An integrated learning approach consisting of multiple decision trees.	1. Applicable to high-dimensional data; 2. Insensitive to outliers. 3. Can be calculated in parallel.	1. Poor model interpretation. 2. High resource consumption.

**Table 2:** Commonly used/mainstream deep learning frameworks.

Major Frameworks	Advantages	Disadvantages	Language	Source Code
Tensorflow	1. It has a powerful computing cluster and can run models on mobile platforms such as iOS and Android; 2. It has better visualization effect of computational graph.	1. Lack of many pre-trained models; 2. Does not support OpenCL.	C++/Python/Java/R, etc.	https://github.com/tensorflow/tensorflow (accessed on 1 February 2024).
Keras	1. Highly modular, very simple to build a network; 2. Simple API with uniform style; 3. Easy to extend, easy to add new modules, just write new classes or functions modeled after existing modules.	1. Slow speed; 2. The program occupies a lot of GPU memory.	Python/R	https://github.com/keras-team/keras (accessed on 1 February 2024).
Caffe	1. C++/CUDA/Python code, fast and high performance; 2. Factory design mode, code structure is clear, readable and extensible; 3. Support command line, Python, and Matlab interfaces, easy to use; 4. It is convenient to switch between CPU and GPU, and multi-GPU training is convenient.	1. Source code modification threshold is high, need to achieve forward/back propagation; 2. Automatic differentiation is not supported.	C++/Python/Matlab	https://github.com/BVLC/caffe (accessed on 1 February 2024).
PyTorch	1. API design is very simple and consistent; 2. Dynamic diagrams and can be debugged just like normal Python code; 3. Its error specification is usually easy to read.	1. Visualization requires a third party 2. Production deployment requires an API server.	C/C++/Python	https://github.com/pytorch/pytorch (accessed on 1 February 2024).
MXNet	1. Support for both imperative and symbolic programming models; 2. Support distributed training on multi-CPU/GPU devices to make full use of the scale advantages of cloud computing.	Interface document mess.	C++/Python/R, etc.	https://github.com/apache/incubator-mxnet (accessed on 1 February 2024).

**Table 3:** Disease Prediction and Survival Prediction Algorithms or Algorithms used for Disease Prediction and Survival Prediction.

Reference	Application Object	AI/ML Technology	Advantages	Disadvantage
gcForest [13]	Prediction of breast cancer subtype	Convolutional neural network, spectral clustering algorithm, inductive clustering technique	High prediction accuracy	Discretization leads to information loss
HOPA_MDA [17]	The miRNA disease association prediction	Higher-order proximity	Performance is further improved on the basis of HOP_MD.	The association between unmarked miRNAs and diseases is difficult to measure
FLNSNLI [21]	Predicting the association between miRNA and disease	Fast linear neighborhood similarity	High-precision performance, less data requirements	Initial miRNA and disease associated data are required
RW-RDGN [64]	Disease gene prediction	network embedding representation, Heterogeneous networks	Excellent performance beyond existing similar methods	Application of heterogeneous disease genes to be developed
GCGCN [66]	Survival prediction of breast cancer and lung squamous cell carcinoma	Graph convolution network	Excellent prediction effect and expansion performance	Large sample size is needed to achieve better prediction results
PAN [67]	Prediction of breast cancer recurrence	Annotation-based networks	Solving the Limitation of Personalized Gene Network	Large consumption of calculation process

**Table 4:** Genome-related algorithms.

Type	Reference	AI/ML Techniques	Application Example	Features
Sequence alignment	SPADIS [67]	Approximation algorithms	SNP dataset analysis	Wide application range. Incomplete data annotations easily lead to large deviations.
Sequence correction	EHMEC [69]	Heuristic algorithms	Polyploid reconstruction haplotype	Minimize the number of errors between DNA reading arrays.
	MiRCAp [70]	Next-Generation Sequencing	DNA sequencing instrument	Correct by forming multiple sequence alignments: delete, insert, and replace errors without requiring large storage space.
Sequence relationship	Needleman-wunsch [71], Smith-waterman [71]	Dynamic programming optimization, Bayesian method, Genetic Algorithms	Comparison of nucleic acid or protein sequences	Identify the homology between proteins to track evolution. Pairs sequences search and compare optimized or closely related fragments.
	BLAST [71], FASTA [71]		Paired sequence alignment and search	Depending on the frequency of amino acid distribution, the local similarity of alignment optimization is approximated directly. It can also be used to discover potential homologues.
	MegaBLAT/CombAlign [71]		Structure-based pairwise comparison	Sequence alignment and search program were derived based on BLAST, showing the relationship between single residues and identifying the similarities and different regions between the alignment proteins.
	MSA [71]		Parallel alignment of multi-genome sequences	Explore the similarity and relationship between sequences and find sequence special motifs.
	SEGA/REGA/mGA/Kenobi/K2 [71]		Tracing the origin of sequence evolution	Evolution or genetic algorithm can be used to trace the origin of sequence evolution.
	MAPPIN [72]	Bipartite graph	Globally aligned multiprotein interaction networks and analysis	The PPI network was analyzed, and the topological structure and function similarity regions between molecular networks of different species were found.
Optimistic algorithm	ReChrome [75]	Convolutional neural networks	Histone analysis and gene expression prediction	Can be applied to any size of genomic data, reducing the number of parameters, not affected by any type of overfitting.
	HOGMCS [76]	High-order graph matching	Molecular Mechanism of Cancer	Improve the accuracy and reliability of miRNA–gene interaction recognition.

**Table 5:** Drug Reuse Algorithm.

Algorithm Type	Mainstream Algorithm	Research Status	Prospect
Supervised learning	Machine learning, DTINet, etc.	AUC (Area Under Curve) value of 0.75	Lack of adequate labels and low label quality limit development
Unsupervised learning	Clustering algorithm, MANTRA, etc.	Accuracy is usually moderate	The new drug disease association, which is lacking in the current understanding of pharmacology, has great prospects
Semi-supervised learning	LapRLS, LPMIHN, NetCBP, etc.	There are many successful cases	Strike a balance between accuracy and universality of new examples, with high research value

Annotation: LapRLS: Laplacian Regularized Least Squares, an algorithm based on least squares and Laplace regularization, commonly used for learning and classification tasks on graph-structured data. LPMIHN: Low-Rank and Positive Matrix Incompletion with Hierarchical Norms, is an algorithm for Low-Rank and Positive Matrix Completion combined with Hierarchical Norms for matrix missing, value filling, and prediction tasks. NetCBP: Network-Constrained Bayesian Personalized Ranking, an algorithm based on network constraints and Bayesian personalized ranking, is commonly used in recommender systems for the task of recommending items based on user behavioral data. AUC is an evaluation metric that measures the quality of a binary classification model, representing the probability that the predicted positive case ranks before the negative case.

**Table 6:** A list of recent papers related to medical image detection(D) and classification(C).

Region	References	Modality	Dimension	Method	Performance
Breast	Mohammed et al. [29] (2018)	X-ray	2D	CNN	DDSM: 99.7% Acc(D)/97% Acc(C)
	Antari et al. [91] (2020)	X-ray	2D	CNN	DDSM: 99.17% Acc(D)/97.5% Acc(C) Inbreast: 97.27% Acc(D)/95.32%Acc(C)
	Sekhar et al. [36] (2022)	X-ray	2D	TL, CNN	DDSM: 100% AUC(C) Inbreast: 99.94% AUC(C) MIAS: 99.93% AUC(C)
Lung	Khosravan et al. [93] (2018)	CT	3D	DCNN	LUNA: 0.897 CPM(D)
	Zheng et al. [92] (2020)	CT	2.5D	MIP, CNN	LIDC: 92.7% Sen/1 FPs(D)
	Liu et al. [31] (2021)	CT	3D	MTL, CNN	LUNA: 0.939 CPM(D)

Annotation: DCNN: Deep Convolutional Neural Network, a neural network structure built with multiple convolutional and pooling layers, commonly used for image recognition and computer vision tasks. MIP: Mixed Integer Programming, a mathematical optimization method for optimization problems with integer variables, widely used in combinatorial optimization and decision support systems. CNN: Convolutional Neural Network, a special type of neural network structure that recognizes images and features through convolutional and pooling layers, widely used in the fields of computer vision and natural language processing. MTL: Multi-Task Learning, a machine learning paradigm that improves the generalization performance and effectiveness of a model by learning multiple representations of related tasks simultaneously. TL: Transfer Learning, transferring knowledge learned on one task to another related task to speed up learning and improve model performance.

**Table 7:** A list of recent papers related to medical image segmentation (“Private” denotes private dataset).

Region	References	Modality	Dimension	Method	Performance
Breast	Singh et al. [98] (2020)	X-ray	2D	GAN, CNN, Semi-supervise	DDSM: 94% DSC, 87% IoU
	Essam et al. [102] (2021)	Infrared Image	2D	MH, ML	Private. It is better than the other nine metaheuristics (MH).
Brain tumor	Yu et al. [95] (2021)	MRI	3D	CNN	BRATS2018: 86.45% mDSC, 3.67 mHD BRATS2019: 84.61% mDSC, 3.69 mHD
Vessel	Gur et al. [101] (2019)	Microscopic Image	2D	Unsupervised-DL, Morphology	VessINN: 82.9% DSC, 99.2% Sen DeepVess: 77.6% DSC, 92.3% Sen
	Feng et al. [99] (2021)	Pathological Image	2D	GAN	Private: 99.5% DSC, 97.25% mIoU
	Arias et al. [96] (2021)	OCT	2D	CNN	DRIVE: 89.97% Sen, 96.90% Spe, 95.63% Acc
Lung	Shakibapour et al. [100] (2018)	CT	3D	CNN, Unsupervise	LUNA: 82.35% DSC LIDC: 71.05% DSC
	Wu et al. [37] (2020)	CT	3D	CNN, CRF	LIDC: 83.3% DSC
	Usman et al. [97] (2020)	CT	2.5D	Semi-Automatic, CNN	LIDC: 87.5% DSC

**Table 8:** Medical Image Registration in Recent Years.

Region	Reference	Modality	Method	Contributions
Lung	Cai, et al. [103] (2021)	MRI	Landmark-based	Adaptive landmark weighting strategy can reduce the error caused by landmark mismatch.
	Hansen and Heinrich [104] (2021)	CT	GCN and CNN	CNN and GCN are used to extract discrete displacement space and spatial dimension
	Xue, et al. [38] (2019)	CT	MRF-based	Design a higher-order energy function to maintain the topology.
Brain	Huang, et al. [105] (2021)	MRI	STN-based	(1) Integration of multimodal affine and deformable transformations. (2) Derivation of reversible deformation.
	Huang, et al. [106] (2021)	MRI	DNN-based	(1) A new multi-scale cascade network. (2) Design a difficulty sensing module to gradually feed forward the hard region to subsequent subnetworks.
	Fan, et al. [107] (2019)	MRI	FCN-based	(1) Use deformation field to guide ground truth. (2) Use the difference between the images after registration to guide the image heterogeneity.
Prostate	Fu, et al. [108] (2021)	US and MRI	CNN-Based	Combine with point cloud matching for registration.
	Sood, et al. [109] (2021)	MRI and Histopathology images	GAN-based	(1) Introduce a new super-resolution generative adversarial network. (2) Don’t require interpolation.

**Table 9:** Recent research on EHR data-assisted patient representation learning.

Type	Reference	Method	Deficiency
Patient representation based on discrete medical concepts	[45]	Clustering and association rules	Negative detection and word sense disambiguation in the model may make some symptom concepts missed.
Patient representation based on time series medical data	[41]	Improved BERT model	The extracted semantic features may affect the performance of the model due to the constraints of the bag-of-words assumption.
	[42]	Attention-based predictive model	Model does not take the alignment of ICD codes in different standards into account.
	[118]	Temporal tree model	Obtained patient representations may only perform well on upstream tasks such as computing similarity.
Patient representation based on multimodal data	[117]	Clustering and CNN	Models account for imputation on incomplete datasets, but also introduce data bias and noise.
	[12]	CNN-LSTM	Models are difficult to interpret and may not be clinically acceptable.
	[119]	Collective Hidden Interaction Tensor Decomposition Model	The process of reconstructing hidden interaction tensors to infer unobserved modes is difficult to explain.

**Table 10:** Comparison of technical means of privacy protection.

References	Technical Tools	Protection Links			Description
		Transmission	Storage	Visualization	
[50]	Game Theory	√			Proposing a Markov theory-based game model for privacy protection in e-health applications.
[51]	Blockchain	√	√		Proposing a blockchain technology-based authentication scheme for securing medical data.
[132]	Anonymous communication protocol	√			Proposing an anonymous communication protocol for mobile health to protect data security and identity privacy.
[133]	Visual Encryption			√	An overview of steganography and visual cryptography for more than 30 models.
[134]	Federal Learning	√	√		Proposing a federal learning framework that does not transmit medical data but only the parameters of the training results.

**Table 11:** Common classification of medical robots.

Classification	Application Type	Application Example
Surgical robots	Orthopaedic robots	Pelvic fracture repositioning robot
	Surgical robots	Automatic suture surgical robot, soft tissue surgery robot.
Rehabilitation robots	Rehabilitation training robots	Portable planar passive rehabilitation robot; ankle rehabilitation robot.
	Exoskeleton robots	Upper limb rehabilitation exoskeleton robot.
Assistive robots	Positioning and diagnostic robots	Spinal injection needle positioning robot; soft surgery robot for lung cancer diagnosis and treatment; airbag endoscopy robot; magnetic positioning robot.
	Robot assistant	MRI-guided low back pain injection with a fully driven body robotic assistant.
	Telemedicine robots	Telemedicine robot based on standard imaging technology robotic arm control.

**Table 12:** Summary of the literature application in this study.

Literature	Specific Application Areas
References [13,15,17,21,62,63,64,65,66,67,68,69,70,71,72,73,74,75,76,77,78,79,166]	Genomics
References [23,24,25,26,27,28,76,77,78,79,80,81]	Drug Discovery
References [3,4], References [6,14,16,19,20,29,30,31,33,35,36,37,38,82,84,85,86,87,88,89,90,91,92,93,94,95,96,97,98,99,100,101,102,103,104,105,106,107,108,109,110,111,112,113,114,115,130,149,151,153,155,156,157,159,161]	Medical image
References [41,42,43,44,45,50,116,117,118,119,120,121,122,123,124,154]	Electronic health Record
References [46,47,48,51,52,59,60,61,125,126,127,128,129,131,135,136,137,138,140,167]	Health Management
References [53,54,55,141,142,143,144,145,146,147,164]	Medical robots
References [1,12,168]	Artificial intelligence medical assisted teaching
References [5,7,9,10,12,22,53,130,139,160,162,163,165,169]	Comprehensive review
References [8,132,133,134]	Medical data security
References [11,61]	Medical AI models and frameworks
References [2,18,24,32,34,39,40,49,56,57,58,83,148,150,152,158,164,170,171]	Other
